# Choice architecture interventions to change physical activity and sedentary behavior: a systematic review of effects on intention, behavior and health outcomes during and after intervention

**DOI:** 10.1186/s12966-020-00942-7

**Published:** 2020-04-07

**Authors:** Lorraine L. Landais, Olga C. Damman, Linda J. Schoonmade, Danielle R. M. Timmermans, Evert A. L. M. Verhagen, Judith G. M. Jelsma

**Affiliations:** 1 Department of Public and Occupational Health, Amsterdam UMC, Vrije Universiteit Amsterdam, Amsterdam Public Health research institute, Van der Boechorststraat 7, NL-1081 BT Amsterdam, The Netherlands; 2 Medical Library, VU University Amsterdam, Amsterdam, The Netherlands; 3 Department of Public and Occupational Health, Amsterdam Collaboration on Health & Safety in Sports, Amsterdam Movement Sciences, Amsterdam UMC, Vrije Universiteit Amsterdam, Amsterdam, The Netherlands

**Keywords:** Choice architecture, Nudging, Behavioral economics, Environmental intervention, Health behavior, Physical activity, Sedentary behavior, Public health

## Abstract

**Background:**

Choice architecture interventions, which subtly change the environment in which individuals make decisions, can be used to promote behavior change. This systematic review aimed to summarize studies on micro-environmental choice architecture interventions that encouraged physical activity or discouraged sedentary behavior in adults, and to describe the effectiveness of those interventions on these behaviors – and on related intentions or health outcomes – in presence of the intervention and after removal of the intervention (i.e. post-intervention, regardless of the time elapsed).

**Methods:**

We systematically searched PubMed, Embase, PsycINFO and the Cochrane Library for (quasi) experimental studies published up to December 2019 that evaluated the effect of choice architecture interventions on physical activity and sedentary behavior, as well as on intentions and health outcomes related to physical activity/sedentary behavior. Studies that combined choice architecture techniques with other behavior change techniques were excluded. All studies were screened for eligibility, relevant data was extracted and two independent reviewers assessed the methodological quality using the QualSyst tool.

**Results:**

Of the 9609 records initially identified, 88 studies met our eligibility criteria. Most studies (*n* = 70) were of high methodologic quality. Eighty-six studies targeted physical activity, predominantly stair use, whereas two studies targeted sedentary behavior, and one targeted both behaviors. Intervention techniques identified were prompting (*n* = 53), message framing (*n* = 24), social comparison (*n* = 12), feedback (*n* = 8), default change (n = 1) and anchoring (n = 1). In presence of the intervention, 68% of the studies reported an effect of choice architecture on behavior, whereas after removal of the intervention only 47% of the studies reported a significant effect. For all choice architecture techniques identified, except for message framing, the majority of studies reported a significant effect on behavioral intentions or behavior in presence of the intervention.

**Conclusions:**

The results suggest that prompting can effectively encourage stair use in adults, especially in presence of a prompt. The effectiveness of the choice architecture techniques social influence, feedback, default change and anchoring cannot be assessed based on this review. More (controlled) studies are needed to assess the (sustained) effectiveness of choice architecture interventions on sedentary behavior and other types of physical activity than stair use.

## Introduction

An important public health challenge of the twenty-first Century is to increase individuals’ levels of physical activity and to reduce their sedentary behavior. One third of the adult population worldwide does not reach the public health guidelines for recommended levels of physical activity [1], and almost one in five Europeans report sitting more than 7.5 h per day [2]. This is worrisome, given that physical inactivity and excessive sedentary behavior independently increase the risk of non-communicable diseases, and can shorten life expectancy [3–7].

A promising approach to break individuals’ unhealthy habits and promote healthy behavior (e.g. increase physical activity and decrease sedentary behavior) is to make subtle changes to the micro-environment in which individuals make decisions, an approach termed ‘choice architecture’ or ‘nudging’ [8–12]. The micro-environment refers to relatively small settings, such as homes and workplaces [13, 14]. Choice architecture is built on the principle that human decision making is often based on automatic and/or heuristic thought processes, rather than effortful deliberate processes alone [15–18]. These automatic thought processes play a considerable role in daily behavior, including habits [19]. Habits are context-response associations in memory that develop as individuals repeat behavior in daily life [9]; once a habit is formed, merely perceiving a certain context can automatically trigger the associated behavioral response [17].

Choice architecture interventions are applied in the physical, social and/or information environment [10, 15, 20]. In the physical environment, for instance, individuals can be prompted to take the stairs instead of the elevator through footprints on the floor that lead to the stairwell [21]. An example of an intervention in the social environment is the use of social norms, which can be either descriptive (i.e. providing information about the behavior of others) or injunctive (i.e. providing information about others’ approval) [22]. Finally, the information environment includes interventions that alter the way in which messages are presented or framed, for example in terms of gains (i.e. emphasizing the benefits of the desired behavior) or losses (i.e. emphasizing consequences of the undesired behavior) [11, 20, 23, 24].

In recent decades, choice architecture has gained momentum in the field of public health and health promotion [10, 12, 25, 26]; however, its theoretical principles originate in a long tradition of judgment and decision making research [16, 20, 27]. Past research has demonstrated that choice architecture interventions can effectively change behavior in a variety of health domains [10]; however, studies on choice architecture in the domain of physical activity and sedentary behavior have received relatively little attention compared to, for example, dietary behavior (e.g. [28–30]). The current review will therefore focus on choice architecture in the domain of physical activity and sedentary behavior.

Two scoping reviews have previously provided an overview of studies using choice architecture interventions to promote physical activity [14, 31], though both reviews only sparsely reported on the effectiveness of the interventions on physical activity. Moreover, there is still a lack of insight regarding the extent to which choice architecture interventions can effectuate durable behavior change after removal of the intervention [15]. It is important to make a distinction between initial behavior change and maintenance of behavior change [32], especially since interventions that effectuate behavior change during the intervention often fail to maintain this change in the long term after removal of the intervention [8, 9]. Finally, a more extensive insight into the effectiveness of choice architecture interventions could be obtained by looking at changes in behavioral intentions and health outcomes related to physical activity and sedentary behavior. It should be noted, however, that changes in intentions do not always equate to changes in behavior [33].

The aim of the current systematic literature review is therefore to summarize studies on micro-environmental choice architecture interventions that encourage physical activity or discourage sedentary behavior in adults, and to describe the effectiveness of those interventions on these behaviors – and on related intentions or health outcomes – in presence of the intervention and after removal of the intervention (i.e. post-intervention, regardless of the time elapsed).

## Methods

This systematic literature review was reported in accordance with the Preferred Reporting Items for Systematic Reviews and Meta-analyses (PRISMA) guidelines [34]. The review was prospectively registered with the International Prospective Register of Systematic Reviews (PROSPERO) on October 26, 2018 (PROSPERO 2018: CRD42018102999).

### Definitions

For the purpose of this review, choice architecture interventions were defined as *interventions that alter the presentation of a choice through information or through the physical or social micro-environment in which individuals make decisions, with the intention of changing health-related choices and behaviors*. This definition was based on the descriptions of choice architecture by Hollands et al. (2013), Thaler and Sunstein (2008) and Münscher et al. (2016) [10, 11, 14]. In addition, we specified three types of environments in which choice architecture interventions can be applied: the physical, social and information environment. We did not consider interventions that (a) are conducted in the macro-environment, such as the construction of parks and bicycle paths in a city, (b) limit freedom of choice, such as mandates, (c) make use of economic instruments, such as financial incentives, (d) have commercial purposes or (e) solely aim to raise awareness [10, 14].

The outcome measures of interest were (a) the intention or motivation to be physically active/less sedentary; (b) behavioral measures of physical activity or sedentary behavior; and (c) anthropometric and cardiovascular health outcomes (e.g. change in body weight and blood pressure). Outcomes could be self-reported, measured by wearable health monitoring devices, or assessed through biometric measurements.

### Search strategy

In collaboration with a medical librarian (LS), a comprehensive search was performed in the bibliographic databases PubMed, Embase, PsycINFO (via Ebsco) and the Cochrane Library from inception to December 13, 2019. Search terms included controlled terms (MeSH in Pubmed, Emtree in Embase and thesaurus terms is PsycINFO) as well as free text terms. In terms of *Population, Intervention, Comparison, Outcome* and *Study design* (PICOS), the search strategy included terms for *Intervention* (e.g. ‘choice architecture’), *Outcome* (e.g. ‘health behavior’) and *Study design* (e.g. ‘randomized controlled trial’); *Population* and *Comparison* were manually checked during the article selection phase. Search terms were used as index terms or as free-text words; for most terms, synonyms and closely related words were included. A search filter was used to limit for experimental and quasi-experimental studies. The search was performed without date or language restriction. The full search strategies for all databases can be found in Additional file 1. Retrieved articles were imported in EndNote and subsequently de-duplicated using the Bramer method [35]. Additional references were obtained by hand-searching reference lists of included articles (backward search) and by citation search for included articles (forward search).

### Eligibility criteria

Articles were eligible for inclusion if they (a) investigated the effect of a choice architecture intervention on physical activity or sedentary behavior, the intention to engage in these behaviors and/or associated health outcomes; (b) studied an adult population (aged 18 years and over); and (c) contained an experimental or quasi-experimental study design. To determine whether the studies derived from the search contained a choice architecture intervention, we used the abovementioned operational definition of this term and the taxonomy of choice architecture techniques from Münscher et al. (2016) [11]. Following from this, interventions did not necessarily need to be labeled as ‘choice architecture’ by the original studies. Articles were excluded if (a) they were written in a language other than English; (b) the study population consisted entirely of individuals with a communicable disease, psychiatric disorder or cancer; or (c) a combination of choice architecture and other behavioral change techniques was used, because this would interfere with our aim to attribute the effect to the choice architecture component(s) separately.

### Article selection

Rayyan, an internet-based software program that facilitates collaboration among reviewers, was used for the study selection process [36]. As a first step, this process consisted of screening all titles against the eligibility criteria, which was done by one researcher (LL). Subsequently, abstracts of the remaining articles were screened by two researchers independently. In this phase, one researcher (LL) covered all articles, and two other researchers (JJ, OD) both covered a different half of the articles. The degree of inter-rater agreement was 81.3% for abstract assessments. One researcher (LL) subsequently screened all full-texts against the eligibility criteria. In case of doubt, two other researchers (JJ, OD) were consulted. Disagreements between reviewers were resolved through discussion.

### Data extraction

One researcher (LL) extracted data from the included studies using a standardized form. Extracted data included study design, setting, target behavior, population characteristics, sample size, details of the intervention and comparison condition, intervention technique, type of environment (physical, social and/or information environment), outcome measurement and findings in presence of the intervention and after removal of the intervention. Outcomes were categorized as *in presence of the intervention* if the intervention was present at the moment of measurement, or if the effect was measured directly after exposure to the intervention. Outcomes were categorized as *after removal of the intervention* if the intervention was no longer present at the moment of measurement. An exception to this applied to interventions conducted in the information environment, since these interventions were typically of much shorter duration. For these interventions, the following cut-off points were used: *in presence of the intervention*: measurements directly after exposure to the intervention up to 1 week after exposure to the intervention; *after removal of the intervention*: measurements > 1 week post-intervention.

Intervention effectiveness was determined by the statistical significance of the effect (significant/not significant) as reported by the original studies. Unless otherwise specified, significant effects reported in the current review refer to effects in the healthy direction. For studies with multiple post-intervention measurements, we reported the outcomes of the measurement most distant from the end of the intervention. Studies that reported both significant and not significant effects on the same outcome variable (e.g. a significant effect on physical activity for women, but not for men) were labeled ‘mixed effects’. Note that in this review, significant effects in experimental studies with pre- and post- measures refer to a significant increase in the intervention condition compared to the comparison condition over time (i.e. baseline compared to follow-up), whereas significant effects in studies with a factorial design refer to a significant increase in one condition compared to another condition.

### Quality assessment

The methodological quality assessment served to inform interpretation of findings, rather than to determine study eligibility. Study quality was assessed independently by two researchers (LL and JJ) using the QualSyst tool from Kmet el al. (2004) [37], allowing assessment of both experimental and quasi-experimental studies. The tool consisted of fourteen items to be scored ‘Yes’ [2], ‘Partial’ [1], ‘No’ (0) or ‘Not applicable’ (N/A), depending on the degree to which specific criteria were met or reported. Aspects covered include quality of study design, confounders, blinding, selection bias and misclassification bias. Discrepancies in assessments between reviewers were resolved through discussion. For each study, a summary score was calculated by summing the total score obtained and dividing it by the total possible score. A quality score of ≥.75 indicates strong quality, a score between .55 and .75 moderate quality, and a score ≤ .55 weak quality.

### Data synthesis

High heterogeneity between studies with regard to study design, intervention characteristics, type of outcome measure and outcome measure assessment prevented performance of a meta-analysis. Instead, we synthesized extracted data by narratively summarizing the characteristics, quality and findings of the included studies. After summarizing the content of interventions, one researcher (LL) inductively identified different intervention techniques by checking the intervention components described in the studies against our operational definition of choice architecture and the choice architecture techniques described by Münscher et al. [11]. Techniques reported in the current review were termed in line with the general choice architecture literature as much as possible (e.g. [10]). The synthesis was structured around (a) the choice architecture techniques identified and (b) the effectiveness of interventions in changing intentions, behaviors or health outcomes in presence of the intervention and after removal of the intervention.

## Results

### Study selection

**Fig. 1:**
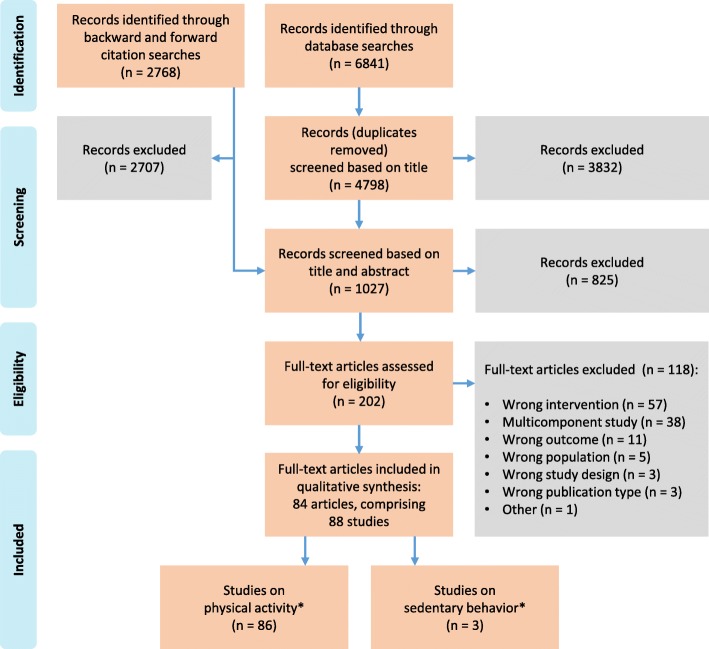
PRISMA flow diagram of the study selection process. * One study measured both physical activity and sedentary behavior [38]

Figure 1 shows the flow diagram of the study selection process. The database searches initially identified 6841 records, of which 4798 remained after removal of duplicates from the database searches. Backward and forward citation searches identified 2768 records. A total of 202 full-text articles were assessed for eligibility. Eighty-four articles were included in this review, comprising 88 unique studies.

### Study characteristics

Table 1 summarizes study characteristics of the included studies. Studies were conducted in the United States (*n* = 38), Europe (*n* = 37), Canada (*n* = 6), China (*n* = 4) or Australia (n = 3). The number of participants across included studies ranged from 30 [47] to 9729 [116].

**Table 1:** Characteristics and key findings of included studies

Author(s), Year, Country	Subtype of intervention	Target behavior, Environment	Study design, Setting	Population description	Intervention description	Outcome measurement, Data collection^**a,b**^	Intention in presence of intervention^**c**^	Behavior in presence of intervention^**c**^	Health outcomes in presence of intervention^**c**^	Intention after removal of intervention^**c**^	Behavior after removal of intervention^**c**^	Quality score
Allais et al. 2017, France [39]	Prompting	PA, PE	QE, Time series design, Metro station	General population, *n* = 205, 62.4% female	Motivational message prompts displayed in 2 metro stations for 3wk to promote stair use (floor stickers & footprints, posters and stair-riser banners). Messages either emphasized ease of stair use (I1) or health benefits (I2). Comparison: 1 metro station without prompts.	Daily observations of stair use. Period: B: 2wk, I: 3wk, F: 3wk		**(+)** I1 (*p* < .01) and I2 (*p* < .05) both increased stair use compared to B			**(+/−)** I1 increased stair use (p < .05); I2 showed no effect compared to B	1.00
Andersen et al. 2013, Denmark [40]	Prompting	PA, PE	E, Pretest-posttest design, E-mail	Office workers, *n* = 160, 78.1% female	Email-based encouragements once a wk. (for 10wk) to walk the stairs for 10 min a day. Comparison: Weekly reminder to continue usual physical activities.	Aerobic fitness (VO_2max_), blood pressure, leisure time PA, weight, body fat percentage. Time points: B & F: 10wk			**(+/−)** Increased aerobic fitness for intervention compared to comparison group (ß 1.5, 95%CI:0.6,2.3); no change in blood pressure, leisure time PA, weight or body fat percentage			1.00
Andersen et al. 2008, USA [41]	Prompting	PA, PE	QE, Pretest-posttest design, Conference center	Health professionals, 16,978 observations, 35.4% female	A motivational sign ‘Be a role model, use the stairs!’ displayed during 1 day	Observations of stair use. Period: B: 1 day, I: 1 day, F: 1 day		**(+)** Increased stair use compared to B (*p* < .001)			**(+)** Stair use remained elevated compared to B (p < .001)	0.82
Andersen et al. 1998, USA [42]	Prompting	PA, PE & IE	QE, Time series design, Shopping mall	General population, 17,901 observations, 59.3% female	Two motivational signs displayed in time series: (I1) emphasized health (4wk); (I2) emphasized physical appearance (4wk).	Observations of stair use. Period: B: 4wk, I1: 4wk, I2: 4wk		**(+)** I1 and I2 both increased stair use compared to B; no difference between I1 and I2				1.00
Avitsland et al. 2017, Norway [43]	Prompting	PA, PE	QE, Time series design, Worksite	Employees, 45,231 observations	I1 (5wk): Footprints on floor directing to the stairs. I2 (4wk): I1 + stair-riser banners with a positive feedback message.	Observations of stair use with infrared counters. Period: Intervention: B: 2wk, I1: 5wk, I2: 4wk, F: 3wk.		**(−)** Stair climbing decreased after I1 and I2 compared to B (*p* < 0.001)			**(−)** Stair climbing did not differ from B	0.79
Bellicha et al. 2016, France [44]	Prompting	PA, PE	QE, Controlled time series design, Worksite	Employees, 36,468 observations, 59% female	I1 (4wk): Directional & motivational signs (emphasizing burning calories). I2 (4wk, 3mths after I1): I1 + colorful stair-riser stickers. Comparison: Different building, no intervention.	Observations of stair use with automatic counters. Period: B: 3wk, I1: 4wk, F1: 3wk, I2: 4wk, F2: 3wk, F3: 3wk (3mths after I), F4: 3wk (7mths after I)		**(+)** Increased stair use for I2 compared to comparison site (ß 4.6, 95%CI:2.3,6.9); I1 did not differ from comparison site (ß 1.1, 95%CI:-1.2,3.4)			**(+)** Increased stair use at intervention site compared to comparison site after 7mths (ß 2.9, 95%CI:0.5,5.4)	0.86
Blake et al. 2008, UK [45]	Prompting	PA, PE	QE, Time series design, Hospital	Patients, employees, general population, 143,514 observations	Posters with different messages were displayed (each 1wk) to promote stair use. Posters emphasized either weight loss, health benefits, family or saving time.	Observations of stair use with infrared counters. Period: B: 1wk, I: 4wk, with 1wk between each poster condition		**(−)** No difference in stair use between intervention period and B				0.82
Blamey et al. 1995, Scotland [46]	Prompting	PA, PE	QE, Time series design, Underground station	General population, 22,275 observations of people	Signs emphasizing health and saving time were displayed between the stairs and escalators during 3wk.	Observations of stair use. Period: B: 1wk, I: 3wk, F1: 2wk, F2: 1wk (4wk after I), F3: (12wk after I)		**(+)** Increased stair use compared to B			**(+)** Increased stair use at 12wk post-intervention compared to B (*p* = .01)	0.73
Bond et al. 2014, USA [47]	Prompting, Feedback	SB, PE	QE, Time series design, Online	Overweight and obese individuals, *n* = 30, 83.3% female	Mobile application consisting of SB monitoring, prompts and feedback. In counterbalanced order, participants received 3 PA break reminders (each 1wk): I1: 3-min break after 30 min SB, I2: 6-min break after 60 min SB, I3: 12-min break after 120 min SB. A green ‘go’ light appeared on dashboard after responding.	SB: SenseWear Mini Armband monitor. Period: B: 1wk, I1: 1wk, I2: 1wk, I3: 1wk		**(+)** SB decreased in I1 compared to B (*p* < .005); I2 compared to B (*p* < .005); I3 compared to B (p < .005)				0.88
Boutelle et al. 2001, USA [48]	Prompting	PA, PE	QE, Time series design, University	General population, 35,475 observations	I1: A sign emphasizing health. I2: I1 + artwork and music in stairwell.	Observations of stair use (3 days per wk). Period: B: 2wk, I1: 4wk, I2: 4wk, F: 4wk		**(+)** Increased stair use during I2 compared to B (p < .01); no difference between I1 and B			**(+)** Increased stair use compared to B (p < .01)	0.77
Brownell et al. 1980, USA, Study 1 [49]	Prompting	PA, PE	QE, Time series design, Shopping mall, train station and bus terminal	General population, 21,091 observations	A sign emphasizing heart health displayed for 2wk to promote stair use in 3 different locations, removed for 2 weeks and displayed again for 2 weeks	Observations of stair use, once a wk. Time points: B1: wk. 1 and wk. 2; I: wk. 3 an wk. 4; B2: wk. 5 and 6; I: wk. 7 and 8.		**(+)** More stair use during the two intervention phases compared to B (p < .001)				0.77
Brownell et al. 1980, USA, Study 2 [49]	Prompting	PA, PE	QE, Time series design, Train station	General population, 24,603 observations	A sign that emphasized heart health displayed during 2wk to promote stair use.	Observations of stair use. Period: B: 5 days, I: 2wk, F1: 2wk, F2: 1wk (4wk after I), F3: 1wk (3mths after I)		**(+)** Increased stair use during intervention period compared to B (p < .001)			**(−)** No difference in stair use 3mths post-intervention compared to B	0.77
Bungum et al. 2007, USA [50]	Prompting	PA, PE	QE, Time series design, University, banks and a parking garage	General population, 2050 observations, 53.5% female	Motivational signs that emphasized health or fitness displayed in 8 different buildings for 2wk to promote stair use.	Observations of stair use. Time points: B, I (2 times), F1 (2wk after I), F2 (4wk after I)		**(+)** Increased stair use compared to B (p < .001)			**(+)** Stair use remained higher 4wk post-intervention compared to B (p < .001)	0.82
Cheung et al. 2008, China [51]	Prompting	PA, PE	E, Cluster randomized trial, Mobile text messages	Primary school teachers, *n* = 52, 78.8% female	Teachers from 3 schools received text messages about PA and SB (3 per wk), leaflets with walking trails and posters with messages to promote stair walking in school (during 6wk). Comparison: 1 school, no intervention.	PA: Pedometer. Time points: B: 5 days, F1: 5 days (6wk after B)		**(+/−)** Increased steps-at-work in intervention compared to comparison (*p* < .001); no difference in steps-off-work (*p* = .27) between intervention and comparison				0.61
Coleman & Gonzalez 2001, USA [52]	Prompting	PA, PE	QE, Time series design, Airport, bank, office building & campus location	General population, 115,153 observations, 51.2% female	Signs displayed near the stairs and escalators in 4 different buildings (i.e. airport, bank, library, office) during 4wk, emphasizing a [1] individual promotional health message or a [2] a family promotional health message.	Observations of stair use, 4 days a wk. Period: B: 4wk, I: 4wk, F: 4wk		**(+/−)** Increased stair use compared to B at the bank and airport; results were mixed for the library and office building (i.e. different outcomes in men and women)			**(+/−)** Stair use remained elevated at the bank and airport, while results were mixed for the library and office building (i.e. different outcomes in men and women)	0.82
Eckhardt et al. 2015, USA [53]	Prompting	PA, PE & IE	QE, Time series design, University	General population, 2997 observations, 80% female	I1: Prompt with a general message: ‘Burn calories. Get healthy’ (2wk). I2: Prompt with a specific message: ‘Walking up stairs burns almost 5 times more calories than riding an elevator’, 2wk.	Observations of stair use. Period: B: 2wk, I1: 2wk, I2: 2wk		**(+)** Increased stair use for I2 compared to B (OR 2.04, 95%CI:1.46,2.84); no difference between I1 and B (OR 1.13, 95%CI:0.84,1.52); increased stair use for I2 compared to I1 (OR 1.57, 95%CI:1.13,2.20)				1.00
Engbers et al. 2007, Netherlands [54]	Prompting	PA, PE	QE, Controlled pretest-posttest design, Worksite	Office workers, *n* = 452, 62.9% overweight, 39.6% female	Prompts: signs, footprints on floor, motivational texts (including poems), PA facts and slim-making mirrors were placed around the stairs and elevators for 12mths. Comparison: Different building, no intervention.	Health: blood pressure, weight, BMI. Time points: B, F1: 3mths, F2: 12mths			**(+/−)** At 12mths, HDL-cholesterol (p < .001) and LDL-cholesterol (p < .001) were reduced, BMI had not changed (*p* = 0.20) and systolic blood pressure was increased compared to comparison (p < .001)			1.00
Engelen et al. 2017, Australia [55]	Prompting	PA, PE	QE, Pretest-posttest design, University	University students and employees, 148,071 observations	In 3 buildings [1–3] arrow-signs and motivational posters with messages emphasizing fitness, time and mental health were displayed during 2wk.	Observations of stair use with infrared counters. Period: B: 2wk, I: 2wk		**(+/−)** Increased stair use in building 1 (OR 1.16, 95%CI:1.09,1.23) and building 2 (OR 1.09, 95%CI:1.03,1.15) compared to B; stair use declined in building 3 (OR 0.75, 95%CI:0.72,0.77) compared to B.				0.91
Eves & Masters 2006, China [56]	Prompting	PA, PE	QE, Pretest-posttest design, Pedestrian transit	General population, 57,801 observations, 48.8% female	A prompt with a message emphasizing health displayed between stairs and travelator during 2wk.	Observations of stair use. Period: B: 2wk, I: 2wk		**(−)** No difference in stair climbing for intervention compared to B (*p* = .29)				0.91
Eves et al. 2012, UK [57]	Prompting	PA, PE	QE, Pretest-posttest design, Worksite	Office workers, 123,934 observations, 49.1% female	A prompt with a message about stair climbing and the Mount Everest and an arrow displayed during 18 days.	Observations of stair use with infrared counters. Period: B: 11 days, I: 18 days		**(+)** Increased stair use compared to B (OR 0.95, 95%CI:0.91,0.98)				0.91
Eves et al. 2012, UK [58]	Prompting	PA, PE	QE, Controlled time series design, Worksite	Employees, 58,206 observations	Worksite 1 (I1): Poster emphasizing calorific expenditure (3wk). Worksite 2 (I2): I1 + stairwell messages emphasizing calorific expenditure (3wk).	Time points: B, F1 (2wk after I). PA: Observations of stair use with infrared counters. Period: B: 1wk, I: 3wk		**(+)** Increased stair use at worksite 1 (I1) (OR 1.24, 95%CI:1.15,1.34) and at worksite 2 (OR 1.52, 95%CI:1.40,1.66) compared to B				0.92
Ford & Torok 2008, USA [59]	Prompting	PA, PE	QE, Time series design, University	University students and employees, 18,389 observations	Four different posters (that rotated daily) with messages that emphasized health, blood pressure, or burning calories displayed during 1wk.	Observations of stair use. Period: B: 1wk, I: 1wk, F: 1wk		**(+)** Increased stair use compared to B			**(+)** Increased stair use compared to B.	0.64
Garland et al. 2018, USA [60]	Prompting	PA, PE	QE, Controlled pretest-posttest design, Home environment	Residents of affordable housing, n = 34, 76.5% female	PODPs were displayed, the stairwells were decorated, music was played and elevator speed was delayed during 15mths. Comparison: Different, no intervention.	PA: Physical Activity Questionnaire, derived from the Block Dietary Data Systems. Health: Height, weight, waist- and hip circumference measurements. Time points: B & F (12–15 mths)		**(+)** Increased stair use at intervention site compared to comparison site (*p* = .03)	**(−)** No difference in BMI or waist-to-hip ratio between intervention group and comparison group (*p* = 0.81)			0.68
Graham et al. 2013, USA [61]	Prompting	PA, PE	E, Cluster randomized trial, Worksite	Employees, *n* = 1356, 63.8% female	Stair use was promoted in 3 buildings during 2 years through motivational messages (humorous, gain-framed), music and art in stairwells, signs and a scale (for body weight). Comparison: 3 other buildings, no intervention.	Observations of stair use with infrared counters + self-reported stair use (questionnaire). Period: B: 20 days, F1: 20 days (2 years after B)		**(+)** More stair use at the intervention sites compared to control sites according to objective data (ß 470, 95%CI:282,659) and self-report data (ß 1.56, 95%CI:0.33,2.79)				0.96
Grimstvedt et al. 2010, USA [62]	Prompting	PA, PE	QE, Time series design, University	Students, employees, 8431 observations	Stair use was promoted in 4 buildings during 3wk through messages emphasizing burning calories and arrow-signs.	Observations of stair use. Period: B: 1wk, I: 3wk, F1: 1wk (2wk after I), F2: 1wk (4wk after I)		**(+)** Increased stair use compared to B (OR 1.65, 95%CI:1.47,1.85)			**(+)** Stair use remained elevated 4wk post-intervention compared to B (OR 1.75, 95%CI:1.51,2.03)	1.00
Hodgin & Graham 2016, USA [63]	Prompting	PA, PE	QE, Controlled posttest only design, College campus	Psychology students, *n* = 167, 48.9% female	Participants were either exposed to a body-widening mirror or a body thinning mirror before they were instructed to go to the 4th floor (choice: stairs/ elevator). Comparison: exposure to standard mirror.	Observations of stair use. Time points: F		**(−)** Stair use was not different for the thinning mirror (OR 0.68, 95%CI:0.23,2.01) or widening mirror (OR 0.64, 95%CI:0.20,2.06) compared to the standard mirror				0.88
Kerr et al. 2004, USA [64]	Prompting	PA, PE	QE, Time series design, Worksite	Employees, *n* = 664, 74.2% female	Four phases: (I1) redecorate stairwell (3,5 years); (I2) adding artwork (3,2 years); (I3) adding motivational signs (2,5 years); and (I4) adding music in stairwell (5 mths)	Observations of stair use with infrared counters. Period: B: 52 days, I1: 18 days, I2: 160 days, I3: 382 days, I4: 72 days		**(+/−)** More stair use during first 3mths of sign intervention (I3) (p < .05) and during last 2mths of music intervention (I4) (*p* < .05) compared to B; I1 and I2 did not differ from B				0.86
Kerr et al. 2001, UK [65]	Prompting	PA, PE	QE, Time series design, Worksite	Employees, 14,982 observations	A poster with a message emphasizing health was displayed for 2wk in building 1 (I1) and for 4wk in building 2 (I2)	Observations of stair use (2 times per wk). Period: B: 2wk, I1: 2wk, I2: 4wk		**(−)** No difference in stair use in building 1 (OR 1.04, 95%CI:0.92,1.18) or building 2 (OR 1.22, 95%CI:0.96,1.55) compared to B				0.86
Kerr et al. 2001, UK, Study 1 [66]	Prompting	PA, PE & IE	QE, Time series design, Train station	General population, 25,319 observations	Stair use was promoted by (I1, 2wk) a poster with a message that emphasized health. Followed by (I2, 2wk) a poster emphasized health + saving time.	Observations of stair use. Period: B: 2wk, I1: 2wk, I2: 2wk		**(+)** Increased stair use during I1 (OR 1.12, 95%CI:1.05,1.20) and I2 (OR 1.22, 95%CI:1.15,1.31) compared to B; stair use increased more in I2 than I1 (OR 1.09, 95%CI:1.02,1.15)				0.86
Kerr et al. 2001, UK, Study 2 [66]	Prompting	PA, PE & IE	QE, Time series design, Shopping mall	General population, 12,588 observations	Stair use was promoted by (I1, 2wk) a poster with a message that emphasized health. The next 2wk, a poster emphasized health + saving time (I2).	Observations of stair use. Period: B: 2wk, I1: 2wk, I2: 2wk		**(+)** Increased stair use during I1 (OR 1.49, 95%CI:1.26,1.76) and I2 (OR 1.39, 95%CI:1.19,1.64) compared to B; no difference between I1 and I2 (OR 0.91, 95%CI:0.78,1.06)				0.86
Kerr et al. 2001, UK [67]	Prompting	PA, PE	QE, Controlled time series design, Shopping mall	General population, 23,934 observations	Stair use was promoted by (I1, 2wk) a poster with a message that emphasized health, followed by (I2, 2wk) I1 + a stair-riser banners with multiple messages. Comparison: Different shopping mall, same poster as I1 during 4wk.	Observations of stair use. Period: B: 2wk, I1: 2wk, I2: 2wk		**(+)** More stair use during I2 than comparison site (OR 2.06, 95%CI:1.48,2.87); more stair use at both sites during I1 (OR 2.18, 95%CI:1.69,2.90)				0.82
Kerr et al. 2001, UK [68]	Prompting	PA, PE	QE, Time series design, Shopping mall	General population, 45,361 observations, 58% female	Stair-riser banners with messages about fitness, health and free and easy exercise were displayed during 12wk.	Observations of stair use. Period: B: 2wk, I: 12wk, F1: 2wk, F2: 2 wk. (8wk after I)		**(+)** Increased stair use compared to B			**(+)** Stair use remained elevated 8wk post-intervention compared to B (OR 1.29, 95%CI:1.14,1.47)	0.91
Kwak et al. 2007, The Netherlands [69]	Prompting	PA, PE	QE, Time series design, Worksite	Employees, 6771 observations, 16.1% women	At two different worksites, posters emphasizing energy balance (between diet and PA) were displayed for 3wk	Observations of stair use (3 days per wk). Period: B: 2wk, I: 3wk, F1: 2wk (1wk after I)		**(+)** Increased stair use compared to B (OR 1.19, 95%CI:1.05,1.34)			**(−)** No difference in stair use between follow-up period and B (OR 1.04, 95%CI:0.98,1.12)	0.95
Lewis & Eves 2012, UK [70]	Prompting	PA, PE	QE, Time series design, Tram station	General population, 38,187 observations, 58.0% female	Phase 1: I1: A poster with a message emphasizing body weight was displayed during 2wk. I2: A message emphasizing calories burned was added to I1 during 6wk. Phase 2: 6wk later, I2 alone was displayed again for 6wk, and I1 was added during last 2wk.	Observations of stair use. Period: B: 2wk for each phase, I1: 2wk for each phase, I2: 6wk (phase 1) + 4wk (phase 2)		**(+)** In phase 1, I2 increased stair use compared to I1 (OR 1.20, 95%CI:1.08,1.33); in phase 2, stair use also increased when both interventions were present, compared to only one (I2) (OR 1.15, 95% CI:1.02,1.29)				0.95
Lewis & Eves 2012, UK [71]	Prompting	PA, PE	QE, Time series design, University	General population, 14,138 observations, 46% female	Phase 1 (I1): In 4 buildings, a poster with a message emphasizing burning calories was displayed in the elevator during 5 days. Phase 2 (I2): An extra poster and an arrow-sign were added to I1 during 8 days.	Observations of stair use. Period: B: 5 days, I1: 5 days, I2: 8 days		**(+)** I2 increased stair climbing relative to I1 (OR 1.30, 95%CI:1.20,1.42); no difference in stair use between I1 and B (OR 0.93, 95%CI:0.85,1.02)				0.91
Lewis & Eves 2011, UK [72]	Prompting	PA, PE	QE, Time series design, Metro station	General population, 23,121 observations, 57.9% female	I1: A poster with a message emphasizing body weight was displayed during 2wk. I2: A message emphasizing calories burned was added to I1 during 6wk.	Observations of stair use, 2 times a wk. Period: B: 2wk, I1: 2wk, I2: 6wk.		**(+/−)** No difference between I1 and B (OR 1.30, 95%CI:0.94,1.80); overall stair use increased throughout I1 and I2 (OR 1.33, 95%CI:1.22,1.44)				0.95
Marshall et al. 2002, Australia [73]	Prompting	PA, PE	QE, Time series design, Hospital	General population, 158,350 observations	A sign emphasizing health and fitness was displayed combined with footprints on the floor (twice for 2wk, (phase 1 & 2), with two weeks in between (B2)).	Daily observations of stair use with infrared counters. Period: B1: 3wk, I: 2 × 2wk, B2: 2 wk., F: 2wk		**(+/−)** Increased stair use in phase 1 compared to B1 (OR 1.05, 95%CI:1.01,1.10); phase 2 did not differ from B1 (OR 0.97, 95%CI:0.93,1.01)			**(−)** Decreased stair use 2wk post-intervention compared to B1 (OR 0.76, 95% CI:0.73,0.79)	0.91
Moloughney et al. 2018, USA [74]	Prompting	PA, PE	QE, Time Series design, Worksite	Employees, 139,304 observations	Phase1 (I1): In 2 buildings PODPs (posters and signage) were displayed. In phase 2 (I2) environmental enhancements (including artwork) in the stairwell were added. Comparison: Different building, only PODPs.	Observations of stair use for 4 days. Time points: B, F1: directly after I1, F2: directly after I2, F3: 1 year after I2 (I1 and I2 were still present)		**(+)** Increased stair use for I1 compared to B (OR 1.36, 95%CI:1.31,1.41) and for I2 compared to I1 (OR 1.31, 95%CI:1.25,1.37); stair use remained elevated at F3 compared to baseline in both buildings (p < .001)				0.86
Müller-Riemenschneider et al. 2010, Germany [75]	Prompting	PA, PE	QE, Time series design, Underground station	General population, 5467 observations, 58% female	Posters with the message “Take Me! Your Stairs!” were displayed during 8wk in 3 underground stations	Observations of stair use. Time points (all 1 h): B, I: wk. 1 and wk. 5, F1: wk. 10 (2wk after I)		**(+/−)** Increased stair use for women (*p* < .001), compared to B, but not for men (p > 0.05)			**(+/−)** Stair use remained higher 2wk post-intervention for women (p < .001), compared to B, but not for men (*p* > 0.05)	0.95
Olander & Eves 2011, UK [76]	Prompting	PA, PE	QE, Time series design, University	Employees, 4279 observations, 49.5% female	I1: A workplace wellbeing day (including posters and leaflets). I2: In 4 buildings, a poster with a message emphasizing burning calories was displayed for 5 days.	Observations of stair use. Period: B: 5 days, I1: 1 day, I2: 5 days (7 days after I1)		**(+)** I2 increased stair climbing compared to B (OR 1.20, 95%CI:1.06,1.37); no difference in stair use between I1 and B (OR 1.02, 95%CI:0.88,1.19)				0.95
Olander et al. 2008, UK [77]	Prompting	PA, PE	QE, Time series design, Train station	General population, 36,239 observations, 56.4% female	I1: Stair-riser banners with a message emphasizing calorific expenditure were displayed. I2: I1 + a poster with the same message.	Observations of stair use (2 days per wk). Period: B: 3.5wk, I1: 10.5wk, I2: 3wk		**(+)** I2 increased stair climbing compared to I1 only (OR 1.36, 95%CI:1.16,1.60); no difference in stair climbing between I1 and B (OR 1.00, 95%CI:0.95,1.05)				0.91
Puig-Ribera & Eves 2009, Spain [78]	Prompting	PA, PE & IE	QE, Time series design, Metro station	General population, *n* = 33,119 observations, 64% female	Stair-riser banners with 3 different messages (2wk each). Messages emphasized: (I1) health, (I2) health + save time, (I3) health; protect heart.	Observations of stair use. Period: B: 2wk, I1: 2wk, I2: 2wk, I3: 2wk, F1: 1wk (2wk after I3)		**(+)** All 3 messages increased stair climbing compared to baseline (I1: OR 1.50, 95%CI:1.27,1.78; I2: OR 1.35, 95%CI:1.13,1.60; I3: OR 1.53, 95%CI:1.30,1.81)			**(+)** Stair climbing remained elevated 2wk post-intervention compared to B (OR 1.22, 95%CI:1.01,1.48)	1.00
Slaunwhite et al. 2009, Canada [79]	Prompting	PA, PE & IE	QE, Time series design, Worksite	University community, *n* = 3339, 52.8% female	Posters were displayed with PA health messages that either emphasized (I1) burning calories, (I2) injunctive norm, (I3) descriptive norm, (I4) norms combined consistently, (I5) norms combined inconsistently.	Observations of stair use. Period: B: 1wk, all interventions: 1wk		**(+/−)** Stair climbing increased for the Injunctive norm (I2) message (p < 0.001) and Norms combined consistently (I4) message (*p* < 0.001) compared to B; the remaining conditions showed no effect compared to B				0.95
Swenson & Siegel 2012, USA [80]	Prompting	PA, PE	QE, Time series design, Worksite	Employees, *n* = 340	Stairwells in the building contained multiple interactive paintings (e.g. world map and storyboard) and signs to promote stair use during 6wk. Comparison: Different building, no intervention	Electronic counts of stair use. Period: B: 2wk, I: 6wk		**(+)** Increased stair use during intervention compared to B (Incidence Rate Ratio (IRR) 2.57, 95%CI:2.35,2.82); no change at comparison site (IRR 1.04, 95%CI:0.90,1.20)				0.77
Vanden Auweele et al. 2005, Belgium [81]	Prompting	PA, PE & IE	QE, Time series design, Worksite	Female employees, 3146 observations	I1 (1wk): Health sign beside elevator and stairs on every level that emphasized fitness and health. I2 (1wk): Employees received e-mail from worksite’s doctor about health benefits of PA.	Observations of stair use, multiple days a wk. Period: B: 1wk, I1: 1wk, I2: 1wk, F1: 1wk (3wk after I2)		**(+)** Stair use increased for I1 and I2 compared to B (p < .001); I2 more effective than I1 (p < .001)			**(−)** No difference in stair use 3 weeks post-intervention compared to B (*p* = .52)	0.77
Webb & Cheng 2010, UK [82]	Prompting	PA, PE	QE, Time series design, Shopping mall	General population, 20,807 observations, 53.1% female	Stair-riser banners with a message emphasizing burning calories were displayed during 5wk.	Observations of stair use (2 days a wk). Period: B: 2wk, I: 5wk		**(+)** Increased stair climbing compared to B (OR 1.28, 95%CI:1.08,1.53)				0.95
Webb & Eves 2007, UK [83]	Prompting	PA, PE	QE, Time series design, Shopping mall	General population, 77,266 observations, 57.5% female	Stair-riser banners with messages about burning calories and heart health were displayed on the stairs from the basement to the first floor during 13wk (intervention). Translational effects were measured from the first to the second floor (same building), where no banners were displayed (translational).	Observations of stair use. Period: B: 2wk, I:wk. 1–4 and wk. 13. F1: 2wk (5wk after I)		**(+)** Stair use increased during wk. 1 to 4 at the intervention site compared to B (OR 2.76, 95%CI:2.44,3.12) and at the translational site compared to B (OR 1.39, 95%CI:1.29,1.49)			**(+)** Stair use remained elevated 5wk post-intervention at the intervention site compared to baseline (OR 1.67, 95%CI:1.44,1.94) and at the translational site compared to baseline (OR 1.15, 95%CI:1.06,1.26)	1.00
Webb & Eves 2007, UK [84]	Prompting	PA, PE	QE, Time series design, Shopping mall	General population, 81,948 observations, 55.5% female	Phase 1 (I1, 3wk): Colorful stair risers in staircase to promote stair use. Phase 2 (I2, 3wk): I1 + messages on stair risers emphasizing heart health. Comparison: different staircase (same building), no intervention.	Observations of stair use. Period: B: 3wk, I1: 3wk, I2: 3wk		**(+)** I2 increased stair climbing compared to B (OR 2.90, 95%CI:2.55,3.29); no difference in stair climbing for I1 (OR 0.88, 95%CI:0.77,1.01) or comparison (OR 0.97, 95%CI:0.87,1.09) compared to B; at the same time, stair climbing increased at the comparison staircase compared to B (OR 1.52, 95%CI:1.34,1.74)				1.00
Webb & Eves, 2005, UK [85]	Prompting	PA, PE & IE	QE, Time series design, Shopping mall	General population, 32,597 observations, 54% female	In phase 1 (I1) 8 stair-riser banners displayed a single message (“Keep fit”). In phase 2 (I2) 8 different messages were used, emphasizing health, free and easy exercise and heart health.	Observations of stair use (2 times a wk). Period: B: 2wk, I1: 2wk, I2: 2wk		**(+)** Increased stair climbing during intervention period (I1&I2) compared to B (OR 2.45, 95%CI:2.14,2.80); no difference between phase 1 (I1) and phase 2 (I2), (OR 0.96, 95%CI:0.88,1.04)				0.91
Arora et al. 2006, USA [86]	Message framing	PA, IE	E, 2 × 2 Factorial design, Experimental setting	General population, *n* = 136, 55% female	Participants received a newsletter that was either gain- or loss-framed and had either high or low credibility. Content: statements about the health effects of PA.	Intention: 1 item on a 8-point scale. Time point: F	**(−)** No effect of framing					0.68
Berenbaum & Cheung 2014, Canada [87]	Message framing	PA, IE	E, Pretest-posttest design, Experimental setting	Female undergraduate students, *n* = 60	Participants received either a gain- or loss-framed advertisement containing a message about the benefits or costs of (not engaging in) PA.	Intention: 2 items. Time points: B, F1 (1wk after I) & registration for open gym session. Self-reported PA: IPAQ (B & F2)				**(−)** No difference between gain-framed group and loss-framed group (*p* = .21)	**(+)** More PA in gain-framed group compared to loss-framed group (p = .02)	0.79
Cho et al. 2018, USA [88]	Message framing	PA, IE	E, 2 × 2 factorial design, Experimental setting	Young adults (18–35 years), *n* = 138, 44.2% female	Participants read either a gain- or loss-framed message describing how a running event either influences individual health (individual appeal) or community health (societal appeal).	Intention: 3 items (7-point scale). Time point: F.	**(+/−)** The gain-framed message resulted in increased intentions compared to the loss-framed message (p < .05); no difference between individual appeal and societal appeal (*p* = .06)					0.73
Cohen et al. 2017, USA [89]	Message framing	PA, IE	E, 2 × 2 Factorial design, Mobile text messages	Obese adults (BMI > 27), *n* = 78, 85.4% female	Participants received text messages on their mobile phone (2 per day for 4wk) that were either gain- or loss-framed and either matched or mis-matched to their motivational orientation.	Self-reported PA (IPAQ) & motivation (URICA scale). Time points: B & F	**(−)** No effect of message frame on motivation to exercise	**(−)** No effect of message frame on exercise behavior				0.71
Daffu-O’Reilly et al. 2017, UK [90]	Message framing	PA, IE	E, 2 × 2 Factorial design, Experimental setting	British South Asians, *n* = 179, 60.9% females	Participants watched a short movie in which PA health messages were either gain- or loss-framed and either culturally sensitive (about Asians) or non-culturally sensitive.	Intention: 3 items. Self-reported PA: short form IPAQ. Time points: B & F1: (8wk after I)				**(−)** No effect of message framing (*p* = .71)	**(−)** No effect of message framing (*p* = .73)	0.92
De Bruijn et al. 2014, The Netherlands [91]	Message framing	PA, IE	E, 2 × 2 Factorial posttest-only design, Experimental setting	General population, *n* = 317, 61.7% female	Participants received a leaflet with a PA health message that was either gain- or loss-framed and varied in type of kernel state (attained outcome vs. avoided outcome).	Intention: 2 items (6-point scale). Time point: F	**(−)** No effect of framing (*p* = .43) or type of kernel state (*p* = .94)					0.71
Gray et al. 2011, USA [92]	Message framing	PA, IE	E, 2 × 2 Factorial design, Experimental setting	College students, *n* = 345, 66.4% female	Participant received a PA health text message that was either gain- or loss-framed and either narrative or statistical.	Intention: 2 items (7-point scale). Time point: F	**(+/−)** The gain-framed message resulted in increased intentions compared to the loss-framed message (*p* < .0001); no difference between narrative and statistical message (*p* = .16)					0.82
Jones et al. 2004, Canada [93]	Message framing	PA, IE	E, 3 × 2 Factorial design, Experimental setting	Psychology students, *n* = 450, 69.8% female	Participants received a PA message that had either a credible or a noncredible source. Next, they read a message that was either gain- or loss-framed.	Intention: 3 items. Self-reported PA: subsection Godin Leisure Time Exercise Questionnaire. Time points: B & F1: (2wk after I)				**(−)** No effect for source credibility or for gain/loss-framed messages	**(−)** No effect for source credibility or for gain/loss-framed messages	0.68
Jones et al. 2003, Canada [94]	Message framing	PA, IE	E, 2 × 2 Factorial design, Experimental setting	Psychology students, *n* = 192, 72.4% female	Participants received PA messages that had either a credible or a noncredible source. Next, they read a message that was either gain- or loss-framed.	Intention: 3 items. Self-reported PA: subsection Godin Leisure Time Exercise Questionnaire. Time points: B & F1: (2wk after I)				**(+/−)** Credible source caused more positive intentions than noncredible source (*p* < .03); no effect for gain/loss-framed messages	**(−)** No effect for source credibility or for gain/loss-framed messages	0.71
Kozak et al. 2013, USA [95]	Message framing	PA, IE	E, Pretest-posttest design, Experimental setting	Undergraduate students, *n* = 64, 82.8% female	Normal weight and overweight/obese participants received either gain- or loss-framed messages.	Self-reported PA: sheets to record PA. Time points: B, F: wk. 2		**(−)** No differences in PA after 2wk compared to B for gain or loss-framed messages				0.71
Latimer et al. 2008, USA [96]	Message framing	PA, IE	E, Pretest-posttest design, Experimental setting	Sedentary adults, *n* = 169, 76.1% female	Participants received gain-, loss-, or mixed-framed (control) messages on 3 occasions (B, wk. 1, wk. 5).	Intention: 1 item (5-point scale). PA: IPAQ short form. Time points: B, wk. 2, wk. 9	**(+)** The gain-framed message resulted in increased intentions compared to the loss-framed message (p < .05) at 2 wk	**(−)** No difference in PA between gain-framed and loss-framed group at 2wk		**(−)** No difference between gain-framed and loss-framed group at 9wk	**(+)** Higher PA in gain-framed compared to loss-framed group (p < .05) at 9wk	0.86
Li et al. 2017, China [97]	Message framing	PA, IE	E, Pretest-posttest design, Experimental setting	Sedentary older adults (> 60 years) with T2D, *n* = 211, 52% female	Participants received a pamphlet with either gain- or loss-framed PA messages about physical, psychological and social effects.	PA: accelerometer & daily activity log. Period: F1: 2wk (2wk after I)					**(−)** No difference between gain-framed and loss-framed group	0.71
Li et al. 2013, China [98]	Message framing	PA, IE	E, Pretest-posttest design, Experimental setting	Younger (age range: 18–35) and older (> 65 years) adults, n = 211, 68% female	Participants received a pamphlet with either gain- or loss-framed PA messages about physical, psychological and social effects.	PA: accelerometer & IPAQ daily activity log. Period: F1: 2wk (2wk after I)					**(+)** Increase in accelerometer-monitored PA in gain-framed compared to loss-framed group (p < .05)	0.79
Lithopoulos & Young 2016, Canada [99]	Message framing	PA, IE	E, Pretest-posttest design, Experimental setting	General population, *n* = 176, 59.7% female	Participants were shown gain-framed messages about sports. Comparison: Participants completed a 13-item PA quiz.	Intention: 5 items (7-point scale). Time points: B, F1 and F2 (4wk after I)	**(−)** No difference between gain-framed message and quiz-group					0.79
McCall & Martin Ginis 2004, Canada [100]	Message framing	PA, IE	E, Pretest-posttest design, Experimental setting	Cardiac patients, n = 60, 8.3% female	Participants read either gain- or loss-framed messages that emphasized PA and heart disease Comparison: No messages.	PA: Attendance at a patient exercise program. Period: 3mths (3 mths after I)					**(−)** No difference between gain-framed and loss-framed group (p > .05) or between gain-framed and control group (*p* = .05)	0.71
Morris et al. 2016, UK [101]	Message framing	PA, IE	E, 2 × 2 Factorial design, Experimental setting	General population, *n* = 596, 33% female	Participants received PA messages that were either affective or cognitive and either about short or long term effects. Comparison: Gain-framed PA messages.	Self-reported PA: Godin Leisure Time Exercise Questionnaire. Time points: B & F (1wk)		**(−)** No effect of intervention compared to comparison				0.68
Notthoff et al. 2016, The Netherlands [102]	Message framing	PA, IE	QE, One-group posttest only design, Experimental setting	Older adults, n = 53, 53% female	Participants watched 6 films (in random order) about different physical activities that were either gain- or loss-framed.	Intention: 1 item (5-point scale). Time point: F	**(−)** No difference between gain- and loss-framed messages (*p* = 0.10)					0.95
Notthoff & Carstensen 2014, USA, Study 1 [103]	Message framing	PA, IE	E, Pretest-posttest design, Experimental setting	Younger (M = 21.4 years) and older (M = 74.8) adults, *n* = 126, 59.9% female	Participants received PA messages that were either gain- or loss-framed. Comparison: Neutrally framed messages.	PA: Pedometer. Period: F: 1wk		**(+/−)** Higher step count for gain-framed than for loss-framed messages in older adults (p = .03); no difference between messages in younger adults				0.79
Notthoff & Carstensen 2014, USA, Study 2 [103]	Message framing	PA, IE	E, Pretest-posttest design, Experimental setting	Older adults (M = 75.8 years), n = 59, 79.7% female	Participants received PA messages that were either gain- or loss-framed once per wk. during 4wk.	PA: Pedometer. Period: F: 4wk		**(+)** Higher step count for gain-framed messages than for loss-framed messages (*p* = .04)				0.79
Ratcliff et al. 2019, USA [104]	Message framing	PA, IE	E, 2 × 4 Factorial design, Experimental setting	General population, *n* = 1039, 50.3% female	Participants read messages about the health consequences of physical (in) activity that were either gain- or loss-framed and varied in message dose (i.e. 1, 2, 3 or 4 messages).	Intention: 3 items (5-point scale). Time point: F	**(+/−)** No difference between gain- and loss-framed messages on intention (*p* = .66); the four-dose condition invoked increased intention compared to the one-dose condition (*p* = .04)					0.79
Vanroy et al. 2019, Belgium [105]	Message framing	PA, IE	QE, Pretest-posttest design, Assisted living facilities	Residents of assisted living facilities (65+ years), *n* = 111, 67.3% female	In all conditions, participants received a 3 wk. exercise program with instructions in weekly (1 h) meetings. In the prevention condition (I1), the benefits of the program were loss-framed (in visual, verbal and symbolic information), whereas they were gain-framed in the promotion condition (I2). Comparison: Neutral messages.	Motivation: 16 items on a 7-point scale. PA: Exercise frequency. Time points: B, I (wk 1 and 2), F (wk 3).	**(−)** No differences in motivation between any two conditions at any point in time (p > .05)	**(−)** No differences in exercise frequency between any two conditions at any point in time (*p* > .05)				0.96
Van ‘t Riet et al. 2010, The Netherlands [106]	Message framing, Feedback	PA, IE	E, Pretest-posttest design, Experimental setting	General population, *n* = 299, 55.1% female	Participants received the Dutch PA recommendations, tailored feedback about their PA level and a persuasive PA health message that was either gain- or loss-framed.	Intention: 1 item (7-point scale). Self-reported PA: IPAQ short version. Time points: B, F (only PA): 3mths after I	**(+)** The gain-framed message resulted in increased intentions compared to the loss-framed message (*p* < .01)				**(−)** No difference between gain- and loss-framed message group after 3mths (*p* = .09)	0.75
Wirtz & Kulpavorapas 2014, USA [107]	Message framing	PA, IE	E, 2 × 2 Factorial posttest only design, Experimental setting	Hispanic adults, *n* = 72, 65.3% female	Participants received PA and diet messages that were either narrative or non-narrative and either gain- or loss-framed.	Intention: 3 items. Time point: F	**(+/−)** Loss-framed messages resulted in increased intentions compared to the gain-framed messages (p < .005); no effect of narrative frame (*p* = .41)					0.64
Zenko et al. 2016, USA [108]	Message framing	PA, IE	E, Posttest-only design, Experimental setting	General population, *n* = 295, 35.9% female	Participants were either asked a high- or low-anchor question about PA. Subsequently, they had to describe either positive/negative experiences (respectively) with exercise.	Intention: 3 items (100-point scale). Time point: F	**(+)** Higher exercise intentions for the high-anchor compared to the low-anchor group (p = .04)					0.71
Cooley et al. 2008, Australia [109]	Social norm, Prompting	PA, SE & PE	QE, Time series design, Worksite	Employees, 62,732 observations	Two posters were displayed consecutively (each 6wk, with 4wk in between) to promote stair use: I1: poster positively emphasized free exercise and health; I2: poster negatively emphasized social norm.	Observations of stair use with infrared counters. Period: B: 3wk, I1: 6wk, F1:4wk, I2: 6wk, F2: 4wk		**(−)** No difference between I1 and B (OR 0.6, 95%CI:0.3,1.1) or between I2 and B (OR 1.0, 95%CI:0.5,1.9)			**(−)** Follow-up after removal of I1 and I2 did not differ from B (OR 0.9, 95%CI:0.5,1.6 and OR 1.1, 95%CI:0.5,2.1, respectively)	0.86
King et al. 2016, USA [38]	Social norm, Feedback	PA & SB, SE & IE	E, Pretest-posttest design, Online	Community-dwelling adults, *n* = 89, 95.3% female	Participants used an application for 8wk that was either (I1) Analytic (PA feedback, tips), (I2) Social (social support, normative feedback, modeling) or (I3) Affective (scheduling, attachment). Comparison: Participants using a control app (on dietary behavior).	PA & SB: accelerometer smartphone data and self-reported on a daily basis. Period: I: 8wk		**(+)** Increased MVPA for I2 app compared to comparison app (p = .01), I1 app (p = .04) and I3 app (p = .03); lower levels of sedentary time for I2 app compared to comparison app (p < .001), I1 app (*p* < .001) and I3 app (p = .02)				1.00
Van Hoecke et al. 2018, Belgium, Study 1 [110]	Social norm, Prompting	PA, SE & PE	QE, Time series design, Worksite	Employees, 5676 observations	In phase 1 (I1, 2wk), stair use was promoted through footprints on the floor. In phase 2 (I2, 1wk), these were supplemented with a health message and in phase 3 (I3, 1wk) general feedback about the number of stair users in the building was added.	Observations of stair use. Period: B (1wk), I1 (2wk), I2 (1wk), I3 (1wk), F: 1wk (6wk after I)		**(+)** Stair use was increased in I2 (p < .001) and I3 (*p* < .001), but not in I1 (p = .06) compared to B			**(+)** Stair use remained elevated 6wk post-intervention compared to B (p < .01)	0.73
Van Hoecke et al. 2018, Belgium, Study 2 [110]	Social norm, Prompting	PA, SE & PE	QE, Time series design, Shopping mall	General population, 12,623 observations	In phase 1 (I1, 1wk), stair use was promoted through footprints on the floor. In phase 2 (I2, 1wk), these were supplemented with a ‘stay-in shape’ poster message and in phase 3 (I3, 1wk) general feedback about the number of stair users was added.	Observations of stair use. Period: B (1wk), I1 (1wk), I2 (1wk), I3 (1wk), F: 1wk (13wk after I)		**(+)** Increased stair use in I2 (p < .001) and I3 (p < .001), but not in I1 compared to B			**(+)** Stair use remained elevated 13wk post-intervention compared to B (p < .01)	0.73
Gorin et al. 2013, USA [111]	Behavioral modeling, Feedback	PA, SE & PE	E, Pretest-posttest design, Home environment	Overweight adults (BMI > 25), *n* = 201, 78.1% female	Participants received a behavioral weight loss treatment (BWL) + a treadmill at home, a TV (viewing time) feedback function, motivational posters and a member at home who served as positive role model during 18mths. Comparison: Participants only received BWL.	PA: Paffenbarger Activity Questionnaire (PAQ). Health: height, weight. Time points: B, I: 6mths, F: 18mths		**(−)** No PA differences between intervention and comparison group at 18mths (*p* = .14)	**(−)** No weight-loss differences between intervention and comparison group at 18mths (*p* = .19)			0.88
Van Calster et al. 2017, Belgium [112]	Behavioral modeling	PA, SE & PE	QE, Time series design, Worksite	Employees, 2458 observations, 36% female	In 2 different buildings [1 and 2], a video of a well-known colleague who chooses the stairs instead of the elevator was displayed during 1wk to promote stair use.	Observations of stair use with Infrared counters. Period: B: 1wk, I: 1wk, F: 1wk		**(+)** Stair climbing increased during the intervention compared to B (p < .001)			**(+/−)** In building 1, stair use remained higher during the post-intervention wk. compared to baseline (p < .01); there was no difference compared to B in building 2 (p = .06)	0.86
Zhang et al. 2015, USA [113]	Behavioral modeling, Social comparison	PA, SE	E, Pretest-posttest design, Online	Graduate students, *n* = 217, 71% female	Participants took part in a basic online program for exercise class participation (13wk), supplemented with either (I1) promotional PA media messages or (I2) an online peer network with 6 anonymous others in which PA class enrollment was visible. Comparison: Basic online program.	PA: exercise class enrollment & self-reported PA (period: I: 13wk)		**(+)** Higher enrollment rates during I2 compared to comparison (p = .02) and during the last 6wk of I2 compared to I1 (*p* < .001); no difference between I1 and comparison (*p* = .08); self-reported PA increased for I2 compared to comparison (*p* = .02); no difference between I1 and comparison (*p* = .74)				1.00
Howie et al. 2011, USA [114]	Competition, Prompting	PA, SE & PE	QE, Controlled time series design, University	College students, 5711 observations	Posters and signs were displayed and combined with competitive challenges to promote stair use. Comparison: Different building, no intervention.	Observations of stair use. Period: B: 1wk, I: 2wk, F: 1wk		**(+)** Higher stair use at intervention site compared to B (p < .001); no change at the control site compared to B (*p* = .28)			**(−)** No difference between stair use 1wk post-intervention and B at the intervention site (*p* = .78) or control site (*p* = .51)	0.82
Patel et al. 2019, USA [115]	Competition, Social comparison, Feedback	PA, SE	E, Pretest-posttest design, Online	Overweight and obese employees, *n* = 602, 29.1% female	During 24 wk., participants either competed weekly in groups of 3 for the highest step count (I1), or collaborated within a team for points; points were lost if a participant did not achieve the step goal (I2). Participants selected a daily step goal, tracked their steps and received daily feedback messages on goal performance. Comparison: Only feedback from the wearable device.	PA: daily step counts measured with a wrist-worn wearable device. Period: B: 1wk, I: 24wk, F: 12wk		**(+)** I1 participants achieved step goals more frequently compared to comparison (ß 0.16, 95%CI:0.14,0.18); I2 participants also achieved step goals more frequently compared to comparison (ß 0.11, 95%CI:0.09,0.12)			**(+/−)** I1 participants achieved step goals more frequently compared to comparison (ß 0.07, 95%CI:0.06,0.09); I2 participants did not achieve step goals more frequently compared to comparison (ß 0.03, 95%CI:0.01,0.04)	1.00
Tullar et al. 2019, USA [116]	Competition, Feedback	PA, SE	QE, posttest-only design, Online	Employees, retirees and dependents, *n* = 9729, 81.0% female	In two institutions, participants chose to participate in a team step-challenge (step competition between teams) or an individual challenge (50,000 steps per week for 5 of the 6-week challenge) during 6wk. Feedback on team standings were provided by wellness managers.	PA: Weekly step counts measured with a pedometer. Time point: F.		**(+)** Higher step counts for team-challenge participants compared to individual-challenge participants (ß 80,714.81, 95%CI:58583.59,102,846.00)				0.95
Zhang et al. 2016, USA [117]	Competition	PA, SE	E, Pretest-posttest design, Online	University students, *n* = 790, 71.4% female	Participants were assigned to one of 4 online conditions with 5 anonymous peers during 11wk for attending exercise classes: (I1) competitive relationships + individual incentives; (I2) supportive relationships + team incentives; (I3) I2 combined with team competition. Comparison (I4): no relationships or individual incentives.	PA: Number of exercise classes attended, registered by class instructors. Period: I: 11wk		**(+)** Attendance rates were higher in I1 compared to B (ß 1.06, 95%CI:0.08,2.04); no differences were found for I2 (ß −0.21, 95%CI:-1.21,0.79), I3 (ß 0.06, 95%CI:-1.43,1.55) or I4 (ß 1.72, 95%CI:-1.38,4.82) compared to B				1.00
Patel et al. 2017, USA [118]	Social comparison, Feedback	PA, SE	E, Pretest-posttest design, Online	Families (2 or 3 adult members), *n* = 200, 56.0% female	During 12wk, families selected a step goal increase, tracked their steps, received daily feedback messages on goal performance and received points as a family; points were lost if a family member did not achieve the step goal. Comparison: Same intervention but without points.	PA: daily step counts measured with Fitbit Flex or Smartphone app. Period: B: 1wk, I: 12wk, F: 12wk		**(+)** Intervention group achieved step goals more frequently compared to comparison (ß 0.26, 95%CI:0.20,0.33)			**(+)** Intervention group achieved step goals more frequently compared to comparison at 12 wk. (ß 0.12, 95%CI:0.05,0.19)	1.00
Strath et al. 2011, USA [119]	Feedback	PA, IE	E, Pretest-posttest design, E-mail	Inactive older adults, *n* = 61, 83% female	Participants received a pedometer during 12wk with either (I1) a 10.000 step goal, (I2) I1 + motivational feedback or (I3): I2 + telephone feedback. Comparison: Participants received standard PA education by email.	PA: Pedometer. Period: 12wk (comparison group: only B: 1wk & F:wk. 12)		**(+)** Higher increase in PA for I2 and I3 compared to comparison and I1 (p < .001)				0.75
Anson et al. 2016, USA [120]	Anchoring, Feedback	PA, IE	E, Crossover design, Online	General population, n = 80, 86.3% female	Participants were assigned a daily step goal of either 5000 or 10,000 steps for 28 days each (in random order), and received feedback on goal achievement. Comparison: Participants from intervention group and participants receiving either a 5000 or 10,000 step goal during 56 consecutive days.	PA: Pedometer. Period: I: 56 days		(+) The 10,000 step goal resulted in a higher number of daily steps compared to the 5000 step goal (p < .05)				0.86
Venema et al. 2017, The Netherlands [121]	Default change	SB, PE	QE, Time series design, Worksite	Employees, *n* = 183, 53.4% female	During 2wk, researchers put all sit-stand-desks in the office at stand-up height and a sign was placed on the desks to ask employees to leave the desk at standing height at the end of the workday.	Intention: 3 items (5-point scale). Time points: B, F. Time points: B, I, F1: 2wk after I, F2: 8wk after I	**(+)** Positive change in intention after intervention compared to B (ß 0.11, 95%CI:0.05,0.26)					0.95

Abbreviations: *PA* physical activity, *SB* sedentary behavior, *PE* physical environment, *IE* information environment, *SE* social environment, *E* experiment, *QE* quasi-experiment, *I* intervention, *B* baseline, *F* follow-up, *min* minute(s), *wk* week(s), *mths* month(s), *OR* odds ratio, *CI* confidence interval, *USA* United States of America, *UK* United Kingdom, *PODP* point-of-decision prompt, *MVPA* moderate to vigorous physical activity, *BMI* body mass index, *T2D* type 2 diabetes

^a^F indicates a follow-up measurement immediately after the end of an intervention; F1, F2, etc. indicate follow-up measurements more distant from the end of an intervention

^b^Period: indication of the length of the measurement or observation period; Time point: indication of the moment of measurement(s), specifying the number of weeks or months since the baseline measurement

^c^**(+)** indicates a significant effect of the (main/most intensive) intervention in the desired direction; **(+/−)** indicates both significant and not significant effects on the same outcome variable; **(−)** indicates no effect of the intervention or an effect in the opposite direction

### Design and setting

Thirty-three studies applied an experimental research design [38, 40, 51, 61, 86–101, 103, 104, 106–108, 111, 113, 115, 117–120]^1^: a pretest-posttest design (*n* = 18), factorial design (*n* = 11), cluster randomized design (*n* = 2), post-test only design (n = 1) or cross-over design (n = 1). The remaining 56 studies used a quasi-experimental design (38, 40–49, 51–59, 61–84, 101, 104, 108, 110, 112, 114, 116, 121)^1^; either a time-series design (*n* = 45), pretest-posttest design (*n* = 7) or post-test only design (*n* = 3). Field experiments were most frequently conducted at the workplace (*n* = 19) (40, 42, 43, 53, 56, 57, 60, 63, 64, 68, 73, 78–80, 108, 110, 112, 116, 121)^1^, followed by public transport locations (*n* = 11) (38, 45, 48, 55, 65, 69, 71, 74, 76, 77)^1^, university campuses (n = 11) [48, 50, 52, 53, 55, 59, 62, 63, 70, 71, 76, 114], shopping malls (*n* = 10) (41, 48, 65–67, 81–84, 110)^1^, hospitals (*n* = 2) [45, 73], and the home environment (*n* = 2) [60, 111]. A total of 23 studies were conducted in a laboratory setting (85–87, 89–103, 105–107)^1^. The remaining studies implemented an intervention through a mobile phone application or website (*n* = 8) [38, 47, 113, 115–118, 120], mobile text messages (n = 2) [51, 89] or e-mail (n = 2) [40, 119].

### Study outcome

Of the included studies, 86 studies targeted physical activity and within these studies, seventeen measured the intention to be more physically active [86, 87, 89–94, 96, 99, 104–108, 122]^1^, 74 measured physical activity behavior [38, 39, 41–53, 55–69, 71–85, 87, 89, 90, 93–98, 100–103, 105, 106, 109–120]^1^ and four measured health outcomes [40, 54, 60, 111]. A total of three studies targeted sedentary behavior, of which one measured the intention to be less sedentary [121] and two measured sedentary behavior [38, 47]; none of the studies measured health outcomes. Individuals’ intentions to become physically active or less sedentary were usually measured by one to three questionnaire items on a 5-, 6- or 7-point scale. Physical activity was assessed with objective measuring devices (*n* = 11), including pedometers and accelerometers, validated questionnaires (*n* = 10), such as the International Physical Activity Questionnaire [123], and other self-report tools (*n* = 7), such as activity logs [97, 98, 105]. Studies that measured stair use (*n* = 48) counted the number of individuals that climbed the stairs within a certain time interval (mostly a few hours a day, during multiple weeks) by using observers (*n* = 36) or automatic (infrared) counters (*n* = 12). Three studies measured enrollment or attendance at exercise classes. Sedentary behavior was either assessed objectively [38, 47], for example by the SenseWear Mini Armband monitor [47], or observed by researchers [121]. Health outcomes were determined through biometric measurements, including body weight and blood pressure. The median duration of interventions was 21 days (range: 1 day to 24 months) and the median period between the end of an intervention and the most distant follow-up measurement 28 days (range: 1 day to 3 months).

### Quality of the included studies

Table 1 presents the summarized quality scores for all studies. The majority of included studies (*n* = 70) were of high methodologic quality. The remaining studies (*n* = 18) were of moderate quality, with the lowest quality score being 0.61 [51]. Most of the moderate quality studies investigated the effectiveness of message framing. The relatively low quality score of these studies was mainly due to lack of blinding of investigators and participants or to lack of report on estimate of variance for the main results. A complete overview of quality ratings on all items can be found in Additional file 2.

### Intervention effectiveness

#### Effectiveness in presence of intervention versus after removal

Overall, the effectiveness of interventions was more often measured in presence of the intervention (*n* = 80) than after removal of the intervention (*n* = 34). For intentions measured in presence of the intervention, four studies reported effective interventions [96, 106, 108, 121], four reported mixed effects [88, 92, 104, 107] and six reported no effect [86, 89, 91, 99, 102, 105]. Among the relatively low number of studies (*n* = 5) that measured intention after removal of the intervention, one reported effectiveness [94], whereas four did not [87, 90, 93, 96].

For behavior, 67.6% of the interventions were effective (38, 40, 41, 43, 45–49, 52, 56–61, 65–70, 73, 75–84, 102, 109, 110, 112–120)^1^, 13.2% showed mixed effects [51, 52, 55, 64, 72, 73, 75, 103] and 19.1% did not show an effect in presence of the intervention [43, 45, 56, 63, 65, 66, 89, 95, 101, 105, 109, 111]. After removal of the intervention, 47.1% of the interventions showed a significant effect (40, 43, 45, 47, 49, 58, 61, 67, 77, 82, 86, 97, 110, 118)^1^, 14.7% showed mixed effects [39, 52, 75, 112, 115] and 38.2% did not show an effect [43, 49, 69, 73, 81, 90, 93, 94, 97, 100, 106, 109, 114]. An explorative analysis of characteristics of the studies that reported a significant effect after removal of the intervention revealed that on average, effective interventions lasted longer (7.3 weeks) than interventions that showed no effect (3.7 weeks). Message-framing studies were excluded from this explorative analysis, since these studies involved one-shot interventions.

Of the four studies that measured health outcomes in presence of choice architecture, one study [40] reported a significant effect on aerobic fitness, but not on other health outcomes; one study [54] reported a reduction in cholesterol levels, but no effect on BMI or blood pressure; and two studies [60, 111] reported no effect.

### Intervention techniques

From the 88 included studies, we derived six different choice architecture intervention techniques, each of which is discussed below. Some intervention techniques were almost always applied in the physical-, social- and or information environment; in these cases, the corresponding environment is specified in parentheses.

#### Prompting (physical and information environment)

Fifty-three studies used prompting (38–84, 108, 110, 114)^1^, most importantly the use of point-of-choice prompts, such as posters, signs, stair-riser banners and directional footprints on the floor to promote stair use. Prompting interventions lasted between 1 day [41] and 3.5 years [64]. Among the 50 studies that looked into the effect of prompting on physical activity in presence of the intervention, 37 (74.0%) reported a significant effect (38, 40, 41, 43, 45, 47–49, 52, 56–61, 65–70, 73, 75–84, 110, 114)^1^, eight (16.0%) reported mixed effects [51, 52, 55, 64, 72, 73, 75, 79] and seven (14.0%) reported no effect [43, 45, 56, 63, 65, 66, 109]. Twenty-one studies measured the effect of prompts on physical activity after removal of the intervention; twelve (57.1%) reported a significant effect (40, 43, 45, 47, 49, 58, 61, 67, 77, 82, 110)^1^, three (14.3%) reported mixed effects [39, 52, 75] and six (28.6%) reported no effect [43, 49, 69, 73, 81, 114].

Prompts consisting of a message differed in the topic emphasized; most prompts emphasized the relationship between physical activity and health (*n* = 25) (38, 41, 44, 45, 47–49, 51, 54, 55, 58, 64–68, 72, 77, 80, 82–84, 108, 110)^1^, caloric expenditure (*n* = 13) [44, 53, 58, 59, 62, 70–72, 76, 77, 79, 82, 83], physical fitness (*n* = 6) (49, 54, 67, 72, 80, 110)^1^ or saving time (n = 6) (44, 45, 54, 65, 77)^1^. The messages showed significant effects on physical activity in 91.7% (11/12) of the studies that emphasized caloric expenditure [44, 53, 58, 59, 62, 70, 71, 76, 77, 82, 83], in 72% (18/25) of the studies that emphasized the relationship between physical activity and health (38, 41, 45, 47–49, 51, 58, 65–67, 77, 80, 82–84, 110)^1^, in 67% (4/6) of the studies that emphasized physical fitness [50, 68, 81, 110], and in 50% (3/6) of the studies that emphasized saving time [46, 66, 78]. In six studies, stair use was prompted by making the staircase more pleasant or attractive, for instance by decorating it with artwork and/or by playing music [48, 60, 61, 64, 74, 80]. Five out of these six studies (83.3%) reported a significant effect on physical activity [48, 60, 61, 74, 80], although it should be noted that some interventions were combined with other choice architecture intervention components. Two studies prompted physical activity through e-mail or mobile phone messages that emphasized the health benefits of physical activity; one reported effectiveness [81] and one mixed effectiveness [51]. One study showed significantly reduced sedentary behavior by prompting physical activity breaks through mobile phone messages [47].

#### Message framing (information environment)

Twenty-four studies compared the effect of a message framed in a certain way with a similar message framed in a different way on individuals’ physical activity intentions and/or behaviors (85–107)^1^. The majority of these studies compared gain-framed messages with loss-framed messages (*n* = 21).

Out of the eleven studies [86, 88, 89, 91, 92, 96, 102, 104–107] that measured physical activity intentions in presence of the intervention, five (45.5%) [88, 92, 96, 106, 107] showed that gain-framed messages were more effective than loss-framed messages. However, two of these studies showing effectiveness was of moderate quality [88, 107]. Among the six studies (88, 94, 95, 102, 104)^1^ that measured physical activity behavior in presence of the intervention, one study (16.7%) [103] demonstrated that gain-framed messages were more effective in changing physical activity.

After removal of the intervention, five studies [87, 90, 93, 94, 96] measured the effect of gain- versus loss-framed messages on intentions and nine studies [87, 90, 93, 94, 96–98, 100, 106] on behavior; none of those studies reported a difference between the effect of gain- and loss-framed messages on intentions and in three of those studies [87, 96, 98], gain-framed messages caused a higher increase in physical activity compared to loss-framed messages.

Other types of framing included for example a credible versus non-credible source message (*n* = 2) [93, 94] or a narrative versus non-narrative message (n = 2) [92, 107]. Among these studies, one [94] reported a significant difference between messages: the credible source resulted in higher exercise intentions than the non-credible source.

#### Social influence (social environment)

Twelve studies used social influence interventions (108–118)^1^, including descriptive social norms (*n* = 4) (108–110)^1^, behavioral modeling (*n* = 3) [111–113], encouragement of competition between individuals or teams (n = 4) [114–117], and facilitation of social comparison through information about the performance of others (n = 3) [113, 115, 118]. All studies providing a descriptive social norm (i.e. messages that specify the prevalence of a specific behavior), except one [109], reported a significant effect on behavior in presence of the intervention. One study found a significant increase in physical activity, as well as a significant decrease in sedentary behavior [38]. Three studies using descriptive social norms also measured physical activity after removal of the intervention (108, 110)^1^. Among these, two studies (110)^1^ reported effectiveness; however, both studies were of moderate quality.

Within the three studies in which behavioral modeling was applied (i.e. demonstration of the desired behavior by another person), two studies (66.7%) [112, 113] reported a significant increase in physical activity in presence of the intervention, whereas one did not [111]. One study also measured the effectiveness after removal of the intervention and reported mixed effects: stair use only remained elevated after removal of the intervention in one of the two intervention buildings [112].

The four interventions that encouraged competition all effectively increased physical activity in presence of the intervention. For two of the interventions, effects were also measured after removal of the intervention [114, 115]; these effects were significant in one study [115]. Finally, the three studies that provided information about physical activity performances of others all reported a significant effect on physical activity during the intervention. Measures after removal of the intervention were performed in two of these studies [115, 118]; the effect was significant in one study [118].

#### Feedback

Feedback was used as an intervention technique in eight studies [38, 47, 106, 115, 116, 118–120]. These interventions consisted of behavioral feedback on one’s level or performance of physical activity [38, 106, 115, 116, 118–120], or on time spent in sedentary behavior [47]; all reported a significant effect on behavior in presence of the intervention. Three studies also measured the effectiveness after removal of the intervention [106, 115, 118]; one study [118] found a significant increase in physical activity and one study found a significant increase in physical activity for one condition, but not for another condition [115].

#### Default change

One study changed the default (i.e. a sit-stand desk was placed at stand-up height instead of sitting height) to encourage sedentary office workers to use the desk in a standing position [121]. The results showed that the intention for stand-up working significantly increased from pre- to post-measure.

#### Anchoring

One study used anchoring to increase daily steps – participants were either assigned a 5000 step goal (low anchor), or a 10,000 step goal (high anchor) – and reported that the high anchor condition resulted in a significantly higher number of daily steps compared to the low anchor condition [120].

## Discussion

### Summary of evidence

The aim of this systematic review was to summarize studies on micro-environmental choice architecture interventions that encourage physical activity or discourage sedentary behavior in adults, and to describe the effectiveness of those interventions on these behaviors – and on related intentions or health outcomes – in presence of the intervention and after removal of the intervention. Within the 88 included studies, six broad choice architecture intervention techniques were distinguished, including – in order of decreasing frequency – prompting, message framing, social influence, feedback, default change and anchoring. In the physical environment, we encountered mostly prompting interventions; in the social environment mostly social influence interventions and in the information environment mostly message framing studies. A great majority of studies targeted physical activity, predominantly stair use, while only three studies focused on reducing sedentary behavior. The results of the review suggest that choice architecture interventions effectively encourage stair use in adults, especially in presence of the intervention. However, since we did not assess effect sizes and only few studies reported follow-up outcomes, it remains unclear how meaningful these increases in stair use are on an individual level.

Consistent with previous research on health behavior change interventions in general [9], a higher proportion of studies reported a significant effect on behavior in presence of the intervention compared to after removal of the intervention. The presence of an intervention likely disrupted habitual behavior [124] (e.g. elevator use) and motivated the choice for a different, healthier option (e.g. the stairs) by bringing existing beliefs (such as ‘taking the stairs is good for my health’) into consciousness, while removal of the intervention probably decreased the salience of beliefs about the healthy option [69]. According to Wood & Neal (2016), behavior change interventions of longer duration tend to be more successful, because they allow for formation of new habits [9]. Indeed, the results of the current review demonstrate that interventions that had lasted longer were most successful in maintaining increases in physical activity after removal of the intervention. This finding should, however, be interpreted with caution since we did not control for other factors (e.g. moment of follow-up measurement). Those findings raise the question: how long should choice architecture interventions generally take to promote habit formation? A study by Kaushal et al. (2015) demonstrated that individuals needed at least six weeks of regular gym workouts to establish new exercise habits [125]; according to a study by Lally et al. (2010), the duration of habit formation varies highly between individuals, ranging from 18 to 254 days [126]. A potential disadvantage of a choice architecture intervention of longer duration in the physical environment could be that individuals become accustomed to it and therefore no longer notice it [39].

A relatively high number of studies that examined social influence as choice architecture technique reported significant changes in behavior, especially in presence of the intervention; eight out of ten studies increased physical activity and the only study that targeted sedentary behavior reported a decrease in sedentary behavior. The descriptive social norm interventions may be effective because people generally fear ostracism and experience a robust need to belong, which drives them to behave appropriately and receive approval [127, 128]. Evidence for the effectiveness of social norm interventions has also been demonstrated in other domains, such as alcohol consumption among college students (e.g. [129]). Due to the limited number of social influence studies identified in the current review, we cannot draw conclusions regarding the most effective type of social influence intervention.

With regard to message framing, our review predominantly identified studies that compared gain-framed messages with loss-framed messages. It should be noted that comparisons between message framing conditions differ from the comparisons that were made in most of the other studies included in this review; in the latter, intervention effects were often compared with ‘no intervention’. As opposed to Gallagher and Updegraff (2012) [24], who reported in their meta-analytic review that gain-framed messages more effectively promoted prevention behaviors (including physical activity) compared to loss-framed messages, we found no favorable effect of gain-framed messages over loss-framed messages on physical activity. This inconsistency in findings can be explained by the fact that more recently published message framing studies were included in our study (i.e. [89, 90, 95, 97, 101]), of which the majority did not report a significant effect on physical activity. For *intentions* to engage in physical activity, the findings of our review did not show a favorable effect of gain-framed over loss-framed messages either.

It is hard to assess the effectiveness of studies that investigated feedback, default change or anchoring as choice architecture technique, because those studies were underrepresented. Moreover, most of the studies that contained feedback also contained another choice architecture technique, which hampered assessment of the effectiveness of feedback alone. Studies on sedentary behavior were underrepresented as well. This can be explained by the fact that, contrary to physical inactivity, the adverse effects of excessive sedentary behavior on health have been fully recognized relatively recently [4, 130].

It must be noted that the choice architecture intervention techniques reviewed are not necessarily new compared to the behavior change techniques (BCTs) described in previous taxonomies of choice architecture and taxonomies of BCTs more in general. For example, some BCTs from the Behavior Change Taxonomy from Michie et al. (2013) (e.g. ‘Restructuring the physical environment’ and ‘Restructuring the social environment’) cover choice architecture techniques that were identified by the current review [131]. In our review, we used terms for choice architecture techniques as they are commonly used in the choice architecture literature (e.g. ‘default change’ and ‘anchoring’), because those terms refer to more specific techniques than the techniques from the taxonomy from Michie et al. [10, 12]. In addition to choice architecture techniques, a wide variety of other BCTs exists [131], such as social support or punishment, but our review did not look at combinations of choice architecture and such other BCTs. Since we excluded multicomponent interventions, we cannot assess whether choice architecture techniques alone, or combined with other BCTs, more effectively change physical activity and sedentary behavior. However, the exclusive focus of our review on choice architecture interventions permits attribution of the effects to specifically those interventions.

### Strengths and limitations

Important strengths of our review are the addition of backward and forward citation searches to the database searches and the assessments of study quality by two independent reviewers. This review also contains several limitations. Firstly, accurate assessment of intervention effectiveness was impeded by the fact that (a) few studies adopted a controlled experimental research design; (b) few studies used objective measurement tools; and (c) we reported the effects of interventions in terms of statistical significance – which is less informative than assessment of effect sizes. Moreover, maintenance of behavior change is hard to assess based on this review, due to the often short-term nature of follow-up measures, and the fact that we reported outcomes in terms of ‘presence or absence of the intervention’, without taking the elapsed time at follow-up into account. High heterogeneity between studies in regard to study design, intervention characteristics, type of outcome measure and outcome measure assessment prevented us from conducting a meta-analysis; therefore, we were limited in comparing the effectiveness of interventions between studies. Since the vast majority of studies measured only stair use, the results cannot be generalized to physical activity as a whole. Another limitation relates to the quality assessment: the majority of studies was considered ‘high’ quality, which is improbable considering other literature reviews on physical activity. Therefore, it may be that we selected a too liberal cut-point for ‘high quality’ and/or that the QualSyst tool lacks sensitivity. Furthermore, despite the extensive search strategy conducted, relevant articles may have been missed. Although this limitation applies to all systematic literature reviews, it may be especially the case for this review because there is no commonly shared operational definition of choice architecture. The term choice architecture may thus cover many different intervention techniques that are termed differently in the literature. We attempted to minimalize those limitations by developing an operational definition of choice architecture and by including different concepts and examples related to choice architecture in our search strategy (e.g. *nudging*, *behavioral economics*, *decision environment*). Furthermore, the initial screening of titles was performed by only one researcher. However, this might not have influenced the results since articles were retained for the next screening phase if the researcher doubted about eligibility. Finally, we did not assess the risk of publication bias [132].

## Conclusions

This systematic literature review extends the work of Forberger et al. (2019) [31] and Hollands et al. (2013) [14] by providing a systematic and comprehensive overview of studies that used choice architecture interventions to encourage physical activity or to discourage sedentary behavior in adults. The results of the current review suggest that *prompting* is a promising choice architecture technique to increase stair use over elevator or escalator use. For prompting, but also for other choice architecture techniques, it seems that intervention effectiveness decreases after removal of the intervention, which may be due to the fact that study participants did not (yet) develop the promoted behavior into a habit. The effectiveness of the choice architecture techniques *social influence*, *feedback*, *default change* and *anchoring* is hard to assess based on this review, since studies using those techniques were underrepresented. Finally, only few studies targeted sedentary behavior or other types of physical activity than stair use, such as active commuting and exercise during leisure time, which highlights the need for additional studies on those behaviors. To allow reliable assessment of behavior change and maintenance of behavior change, future studies must use objective measurement tools and a controlled experimental research design with (long-term) follow-up measures.

## Supplementary information

**Additional file 1.** Search strategy for PubMed, Embase, PsycINFO and the Cochrane Library.

**Additional file 2.** Quality ratings of included studies.

## Data Availability

All data generated or analyzed during this study are included in this published article (and its supplementary information files).

## Abbreviations

B: Baseline
BCTs: Behavior change techniques
BMI: Body mass index
CI: Confidence interval
E: Experiment
F: Follow-up
I: Intervention
IE: Information environment
min: Minute(s)
mths: Months
MVPA: Moderate to vigorous physical activity
OR: Odds ratio
PA: Physical activity
PE: Physical environment
PODP: Point-of-decision prompt
PRISMA: Preferred reporting items for systematic reviews and meta-analyses
QE: Quasi-experiment
SB: Sedentary behavior
SE: Social environment
T2D: Type 2 diabetes
UK: United Kingdom
USA: United States of America
wk: Week(s)

## Footnotes

1 The number of studies reported does not always match the number of citations, because four articles covered two studies.
