# Supplementary material for: Choice architecture interventions to change physical activity and sedentary behavior: a systematic review of effects on intention, behavior and health outcomes during and after intervention
Source: Int J Behav Nutr Phys Act. 2020 Apr 7;17:47. doi: 10.1186/s12966-020-00942-7 (PMC7140383; doi:10.1186/s12966-020-00942-7)
Supplement: Supplementary file 1 — Additional file 1. Search strategy for PubMed, Embase, PsycINFO and the Cochrane Library. [file 12966_2020_942_MOESM1_ESM.docx]

**Additional File 1.** Search strategy for PubMed, Embase.com, PsycINFO (via Ebsco) and the Cochrane Library

### Search History PubMed December 13, 2019

| **Search** | **PubMed Query – December 13. 2019** | **Items found** |
| --- | --- | --- |
| #4 | #1 AND #2 AND #3 | 2061 |
| #3 | (("Clinical Studies as Topic"[Mesh:NoExp] OR "Clinical Trials as Topic"[Mesh] OR "Randomized Controlled Trial" [Publication Type] OR "Randomized Controlled Trials as Topic"[Mesh] OR "Controlled Clinical Trial" [Publication Type] OR "Clinical Trial" [Publication Type] OR "Random Allocation"[Mesh] OR randomized[tw] OR randomised[tw] OR randomization[tw] OR randomisation[tw] OR randomly[tw] OR "Control Groups"[Mesh] OR control[tw] OR controll*[tw] OR "Random Allocation"[Mesh] OR trial*[tw] OR random*[tw] OR "Evaluation Studies as Topic"[Mesh] OR "Evaluation Studies" [Publication Type] OR "Follow-Up Studies"[Mesh] OR "Prospective Studies"[Mesh] OR prospectiv*[tw] OR "Cross-Over Studies"[Mesh] OR crossover[tiab] OR cross-over[tiab] OR arm[tiab] OR arms[tiab] OR experiment*[tw] OR "Non-Randomized Controlled Trials as Topic"[Mesh] OR nonequivalent[tw] OR non equivalent[tw] OR pretest*[tw] OR pre test*[tw] OR posttest*[tw] OR post test*[tw] OR quasi experiment*[tw] OR quasiexperiment*[tw] OR "Interrupted Time Series Analysis"[Mesh] OR time series[tw] OR timeseries[tw] OR factorial stud*[tw] OR factorial design[tw] OR factorial experiment[tw] OR feasibility stud*[tw] OR pilot[tw] OR longitudinal[tw] OR exploratory stud*[tw]) NOT (Editorial[pt] OR Letter[pt] OR Case Reports[pt] OR Comment[pt])) NOT (animals[mh] NOT humans[mh]) | 7103461 |
| #2 | "Life Style"[Mesh] OR "Health Behavior"[Mesh] OR "Sedentary Lifestyle"[Mesh] OR "Exercise"[Mesh] OR lifestyle*[tiab] OR life style*[tiab] OR health behaviour*[tiab] OR health behavior*[tiab] OR healthy behaviour*[tiab] OR healthy behavior*[tiab] OR behaviour change*[tiab] OR behavior change*[tiab] OR behavioural change*[tiab] OR behavioral change*[tiab] OR sedent*[tiab] OR sitting[tiab] OR TV-time[tiab] OR TV viewing[tiab] OR watching TV[tiab] OR television[tiab] OR computer time[tiab] OR computer use[tiab] OR screen time[tiab] OR screen-time[tiab] OR gaming[tiab] OR screentime[tiab] OR video gam*[tiab] OR videogam*[tiab] OR physical activ*[tiab] OR physically active[tiab] OR exercis*[tiab] OR stair use[tiab] OR staircase use[tiab] OR physical inactiv*[tiab] OR physically inactiv*[tiab] OR weight reduction[tiab] OR weight loss[tiab] OR weight management[tiab] | 981422 |
| #1 | choice-architecture*[tiab] OR choice presentation*[tiab] OR nudg*[tiab] OR social-marketing*[tiab] OR "Economics, Behavioral"[Mesh] OR behavioral economic*[tiab] OR behavioural economic*[tiab] OR behavioral design*[tiab] OR behavioural design*[tiab] OR environmental change*[ti] OR environmental cue*[ti] OR environmental intervention*[tiab] OR environmental adaptation*[tiab] OR environmental modification*[tiab] OR environmental alteration*[tiab] OR environmental prompt*[tiab] OR environment intervention*[tiab] OR environment change*[tiab] OR environment cue*[tiab] OR environment intervention*[tiab] OR environment adaptation*[tiab] OR environment modification*[tiab] OR environment alteration*[tiab] OR contextual change*[tiab] OR contextual cue*[tiab] OR behavior setting*[tiab] OR behaviour setting*[tiab] OR behavioral setting*[tiab] OR behavioural setting*[tiab] OR choice environment*[tiab] OR decision environment*[tiab] OR decision structur*[tiab] OR decision information[tiab] OR decision assist*[tiab] OR choice set*[tiab] OR communication format*[tiab] OR information architecture*[tiab] OR default*[ti] OR framing[tiab] OR loss frame*[tiab] OR gain frame*[tiab] OR refram*[tiab] OR social proof*[tiab] | 22696 |

### Search History Embase.com December 13, 2019

| **Search** | **Embase.com Query – December 13. 2019** | **Items found** |
| --- | --- | --- |
| #5 | #4 NOT ('conference abstract'/it OR 'editorial'/it OR 'letter'/it OR 'note'/it OR 'short survey'/it) NOT ([animals]/lim NOT [humans]/lim) | 2719 |
| #4 | #1 AND #2 AND #3 | 3252 |
| #3 | ('clinical trial'/exp OR 'randomized controlled trial'/exp OR 'prospective study'/exp OR 'quasi experimental study'/exp OR 'experimental design'/exp OR 'randomized controlled trial'/exp OR 'controlled study'/exp OR 'randomization'/exp OR 'control group'/exp OR 'evaluation study'/exp OR 'follow up'/exp OR 'crossover procedure'/exp OR 'nonequivalent control group'/exp OR 'time series analysis'/exp OR 'factorial design'/exp OR 'feasibility study'/exp OR 'pilot study'/exp OR 'exploratory research'/exp OR randomiz*:ab,ti,kw,de OR randomis*:ab,ti,kw,de OR randomly:ab,ti,kw,de OR control:ab,ti,kw,de OR controll*:ab,ti,kw,de OR trial*:ab,ti,kw,de OR random*:ab,ti,kw,de OR prospectiv*:ab,ti,kw,de OR crossover:ab,ti,kw,de OR ‘cross-over’:ab,ti,kw,de OR arm:ab,ti,kw,de OR arms:ab,ti,kw,de OR experiment*:ab,ti,kw,de OR ‘nonequivalent control group*’:ab,ti,kw,de OR nonequivalent:ab,ti,kw,de OR ‘non equivalent’:ab,ti,kw,de OR posttesting:ab,ti,kw,de OR pretesting:ab,ti,kw,de OR pretest*:ab,ti,kw,de OR ‘pre test*’:ab,ti,kw,de OR posttest*:ab,ti,kw,de OR ‘post test*’:ab,ti,kw,de OR ‘quasi experiment*’:ab,ti,kw,de OR quasiexperiment*:ab,ti,kw,de OR ‘time series’:ab,ti,kw,de OR timeseries:ab,ti,kw,de OR ‘factorial stud*’:ab,ti,kw OR ‘factorial design*’:ab,ti,kw OR ‘factorial experiment*’:ab,ti,kw OR ‘feasibility stud*’:ab,ti,kw OR pilot:ab,ti,kw OR longitudinal:ab,ti,kw OR ‘exploratory stud*’:ab,ti,kw) | 15223631 |
| #2 | 'lifestyle'/exp OR 'health behavior'/exp OR 'exercise'/exp OR lifestyle*:ab,ti,kw OR ‘life style*’:ab,ti,kw OR ‘health* behaviour*’:ab,ti,kw OR ‘health* behavior*’:ab,ti,kw OR ‘behaviour* change*’:ab,ti,kw OR ‘behavior change*’:ab,ti,kw OR sedent*:ab,ti,kw OR sitting:ab,ti,kw OR ‘TV time’:ab,ti,kw OR ‘TV viewing’:ab,ti,kw OR ‘watching TV’:ab,ti,kw OR television:ab,ti,kw OR ‘computer time’:ab,ti,kw OR ‘computer use’:ab,ti,kw OR ‘screen time’:ab,ti,kw OR screentime:ab,ti,kw OR gaming:ab,ti,kw OR ‘video gam*’:ab,ti,kw OR videogam*:ab,ti,kw OR ‘physical* activ*’:ab,ti,kw OR exercis*:ab,ti,kw OR ‘stair use’:ab,ti,kw OR ‘staircase use’:ab,ti,kw OR ‘physical* inactiv*’:ab,ti,kw OR ‘weight reduction’:ab,ti,kw OR ‘weight loss’:ab,ti,kw OR ‘weight management’:ab,ti,kw | 1279742 |
| #1 | 'social marketing'/exp OR 'behavioral economics'/exp OR ‘choice-architecture*’:ab,ti,kw OR ‘choice presentation*’:ab,ti,kw OR nudg*:ab,ti,kw OR ‘social marketing*’:ab,ti,kw OR ‘behavioral economic*’:ab,ti,kw OR ‘behavioural economic*’:ab,ti,kw OR ‘behavioral design*’:ab,ti,kw OR ‘behavioural design*’:ab,ti,kw OR ‘environment* intervention*’:ab,ti,kw OR ‘environment* change*’:ti OR ‘environment* cue*’:ti OR ‘environment* adaptation*’:ab,ti,kw OR ‘environment* modification*’:ab,ti,kw OR ‘environment* alteration*’:ab,ti,kw OR ‘environmental prompt*’:ab,ti,kw OR ‘contextual cue*’:ab,ti,kw OR ‘contextual change*’:ab,ti,kw OR ‘behavior* setting*’:ab,ti,kw OR ‘behaviour* setting*’:ab,ti,kw OR ‘choice environment*’:ab,ti,kw OR ‘decision environment*’:ab,ti,kw OR ‘decision structur*’:ab,ti,kw OR ‘decision information’:ab,ti,kw OR ‘decision assist*’:ab,ti,kw OR ‘choice set*’:ab,ti,kw OR ‘communication format*’:ab,ti,kw OR ‘information architecture’:ab,ti,kw OR default*:ti OR framing:ti,ab,kw OR ‘loss frame*’:ti,ab,kw OR ‘gain frame*’:ti,ab,kw OR refram*:ti,ab,kw OR ‘social proof*’:ti,ab,kw | 30793 |

### Search History PsycINFO December 13, 2019

| **Search** | **PsycINFO (Ebsco) Query – December 13. 2019** | **Items found** |
| --- | --- | --- |
| S5 | S4 NOT ZZ ("abstract collection" OR "bibliography" OR "chapter" OR "clarification" OR "column/opinion" OR "comment/reply" OR "dissertation" OR "editorial" OR "encyclopedia entry" OR "erratum/correction" OR "interview" OR "letter" OR "obituary" OR "poetry" OR "publication information" OR "review-book" OR "review-media" OR "review-software & other") NOT (PO Animal NOT PO Human) | 765 |
| S4 | S1 AND S2 AND S3 | 960 |
| S3 | DE ("Clinical Trials" OR "Prospective Studies" OR "Experimental Methods" OR "Quasi Experimental Methods" OR "Experimental Design" OR "Experiment Controls" OR "Followup Studies" OR "Program Evaluation" OR "Evaluation" OR "Time Series") OR TI (randomiz* OR randomis* OR randomly OR control OR controll* OR trial* OR random* OR prospectiv* OR crossover OR “cross-over” OR arm OR arms OR experiment* OR “nonequivalent control group*” OR nonequivalent OR “non equivalent” OR posttesting OR pretesting OR pretest* OR “pre test*” OR posttest* OR “post test*” OR “quasi experiment*” OR quasiexperiment* OR “time series” OR timeseries OR “factorial stud*” OR “factorial design*” OR “factorial experiment*” OR “feasibility stud*” OR pilot OR longitudinal OR “exploratory stud*”) OR AB (randomiz* OR randomis* OR randomly OR control OR controll* OR trial* OR random* OR prospectiv* OR crossover OR “cross-over” OR arm OR arms OR experiment* OR “nonequivalent control group*” OR nonequivalent OR “non equivalent” OR posttesting OR pretesting OR pretest* OR “pre test*” OR posttest* OR “post test*” OR “quasi experiment*” OR quasiexperiment* OR “time series” OR timeseries OR “factorial stud*” OR “factorial design*” OR “factorial experiment*” OR “feasibility stud*” OR pilot OR longitudinal OR “exploratory stud*”) | 1376472 |
| S2 | DE ("Lifestyle" OR "Active Living" OR "Lifestyle Changes" OR "Health Behavior" OR "Exercise" OR "Weight Control" OR "Sedentary Behavior" OR "Screen Time") OR TI (lifestyle* OR “life style*” OR “health* behaviour*” OR “health* behavior*” OR “behaviour* change*” OR “behaviour change*” OR sedent* OR sitting OR “TV time” OR “TV viewing” OR “watching TV” OR television OR “computer time” OR “computer use” OR “screen time” OR screentime OR gaming OR “video gam*” OR videogam* OR “physical* activ*” OR exercis* OR “stair use” OR “staircase use” OR “physical* inactiv*” OR “weight reduction” OR “weight loss” OR “weight management”) OR AB (lifestyle* OR “life style*” OR “health* behaviour*” OR “health* behavior*” OR “behaviour* change*” OR “behavior change*” OR sedent* OR sitting OR “TV time” OR “TV viewing” OR “watching TV” OR television OR “computer time” OR “computer use” OR “screen time” OR screentime OR gaming OR “video gam*” OR videogam* OR “physical* activ*” OR exercis* OR “stair use” OR “staircase use” OR “physical* inactiv*” OR “weight reduction” OR “weight loss” OR “weight management”) | 349484 |
| S1 | DE ("Social Marketing" OR "Behavioral Economics") OR TI (“choice-architecture*” OR “choice presentation*” OR nudg* OR “social marketing*” OR “behavioral economic*” OR “behavioural economic*” OR “behavioral design*” OR “behavioural design*” OR “environment* intervention*” OR “environment* change*” OR “environment* cue*” OR “environment* adaptation*” OR “environment* modification*” OR “environment* alteration*” OR “environmental prompt*” OR “contextual change*” OR “contextual cue*” OR “behavior* setting*” OR “behaviour* setting*” OR “choice environment*” OR “decision environment*” OR “decision structur*” OR “decision information” OR “decision assist*” OR “choice set*” OR “communication format*” OR “information architecture” OR default* OR framing OR “loss frame*” OR “gain frame*” OR refram* OR “social proof*”) OR AB (“choice-architecture*” OR “choice presentation*” OR nudg* OR “social marketing*” OR “behavioral economic*” OR “behavioural economic*” OR “behavioral design*” OR “behavioural design*” OR “environment* intervention*” OR “environment* adaptation*” OR “environment* modification*” OR “environment* alteration*” OR “environmental prompt*” OR “environment* intervention*” OR “contextual change*” OR “behavior* setting*” OR “behaviour* setting*” OR “choice environment*” OR “decision environment*” OR “decision structur*” OR “decision information” OR “decision assist*” OR “choice set*” OR “communication format*” OR “information architecture” OR framing OR “loss frame*” OR “gain frame*” OR refram* OR “social proof*”) | 26048 |

### Search History the Cochrane Library December 13, 2019

| **Search** | **The Cochrane Library Query – December 13. 2019** | **Items found** |
| --- | --- | --- |
| #4 | #1 and #2 and #3 | 1296 |
| #3 | (randomiz* OR randomis* OR randomly OR control OR controll* OR trial* OR random* OR prospectiv* OR crossover OR “cross-over” OR arm OR arms OR experiment* OR (nonequivalent NEXT control NEXT group*) OR nonequivalent OR “non equivalent” OR posttesting OR pretesting OR pretest* OR pre-test* OR posttest* OR post-test* OR (quasi NEXT experiment*) OR quasiexperiment* OR “time series” OR timeseries OR (factorial NEXT (stud* OR design* OR experiment*)) OR (feasibility NEXT stud*) OR pilot OR longitudinal OR (exploratory NEXT stud*)): ti,ab,kw (Word variations have been searched) | 1267741 |
| #2 | (lifestyle* OR (life NEXT style*) OR (health* NEXT behaviour*) OR (health* NEXT behavior*) OR (behaviour* NEXT change*) OR (behavior NEXT change*) OR sedent* OR sitting OR “TV time” OR “TV viewing” OR “watching TV” OR television OR “computer time” OR “computer use” OR “screen time” OR screentime OR gaming OR (video NEXT gam*) OR videogam* OR (physical* NEXT activ*) OR exercis* OR “stair use” OR “staircase use” OR (physical* NEXT inactiv*) OR “weight reduction” OR “weight loss” OR “weight management”) : ti,ab,kw (Word variations have been searched) | 146159 |
| #1 | (choice-architecture* OR (choice NEXT presentation*) OR nudg* OR (social NEXT marketing*) OR (behavioral NEXT economic*) OR (behavioural NEXT economic*) OR (behavioral NEXT design*) OR (behavioural NEXT design*) OR (environment* NEXT (intervention* OR change* OR cue* OR adaptation* OR modification* OR alteration*)) OR (environmental NEXT prompt*) OR (contextual NEXT change*) OR (contextual NEXT cue*) OR (behavior* NEXT setting*) OR (behaviour* NEXT setting*) OR (choice NEXT environment*) OR (decision NEXT environment*) OR (decision NEXT structur*) OR “decision information” OR (decision NEXT assist*) OR (choice NEXT set*) OR (communication NEXT format*) OR “information architecture” OR default* OR framing OR (loss NEXT frame*) OR (gain NEXT frame*) OR refram* OR “social proof”):ti,ab,kw (Word variations have been searched) | 7673 |
