# Supplementary material for: Choice architecture interventions to change physical activity and sedentary behavior: a systematic review of effects on intention, behavior and health outcomes during and after intervention
Source: Int J Behav Nutr Phys Act. 2020 Apr 7;17:47. doi: 10.1186/s12966-020-00942-7 (PMC7140383; doi:10.1186/s12966-020-00942-7)
Supplement: Supplementary file 2 — Additional file 2. Quality ratings of included studies. [file 12966_2020_942_MOESM2_ESM.docx]

**Additional file 2.** Quality ratings of included studies

| **Study** | **Year** | **Item 1** | **Item 2** | **Item 3** | **Item 4** | **Item 5** | **Item 6** | **Item 7** | **Item 8** | **Item 9** | **Item 10** | **Item 11** | **Item 12** | **Item 13** | **Item 14** | **Summary score** |
| --- | --- | --- | --- | --- | --- | --- | --- | --- | --- | --- | --- | --- | --- | --- | --- | --- |
| Allais et al. (39) | 2017 | 2 | 2 | 2 | 2 | N/A | N/A | N/A | 2 | 2 | 2 | 2 | 2 | 2 | 2 | 1.00 |
| Andersen et al. (40) | 2013 | 2 | 2 | 2 | 2 | 2 | 2 | N/A | 2 | 2 | 2 | 2 | 2 | 2 | 2 | 1.00 |
| Andersen et al. (41) | 2008 | 2 | 1 | 1 | 0 | N/A | N/A | N/A | 2 | 2 | 2 | 2 | 2 | 2 | 2 | 0.82 |
| Andersen et al. (42) | 1998 | 2 | 2 | 2 | 2 | N/A | N/A | N/A | 2 | 2 | 2 | 2 | 2 | 2 | 2 | 1.00 |
| Avitsland et al. (43) | 2017 | 2 | 2 | 2 | 0 | 2 | N/A | N/A | 2 | 2 | 2 | 0 | 1 | 2 | 2 | 0.79 |
| Bellicha et al. (44) | 2016 | 2 | 2 | 2 | 2 | N/A | N/A | N/A | 2 | 2 | 2 | 2 | 0 | 2 | 1 | 0.86 |
| Blake et al. (45) | 2008 | 2 | 2 | 2 | 0 | N/A | N/A | N/A | 2 | 2 | 2 | 2 | 0 | 2 | 2 | 0.82 |
| Blamey et al. (46) | 1995 | 2 | 1 | 2 | 0 | N/A | N/A | N/A | 1 | 2 | 2 | 0 | 2 | 2 | 2 | 0.73 |
| Bond et al. (47) | 2014 | 2 | 2 | 2 | 2 | 1 | 0 | N/A | 2 | 2 | 2 | 2 | 2 | 2 | 2 | 0.88 |
| Boutelle et al. (48) | 2005 | 1 | 2 | 2 | 0 | N/A | N/A | N/A | 2 | 2 | 2 | 0 | 2 | 2 | 2 | 0.77 |
| Brownell et al. (49) | 1980 | 2 | 2 | 1 | 0 | N/A | N/A | N/A | 2 | 2 | 2 | 0 | 2 | 2 | 2 | 0.77 |
| Bungum et al. (50) | 2007 | 2 | 2 | 2 | 1 | N/A | N/A | N/A | 2 | 2 | 2 | 0 | 1 | 2 | 2 | 0.82 |
| Cheung et al. (51) | 2007 | 2 | 2 | 2 | 1 | 0 | 0 | 0 | 2 | 0 | 2 | 0 | 2 | 2 | 2 | 0.61 |
| Coleman & Gonzalez (52) | 2001 | 2 | 2 | 2 | 1 | N/A | N/A | N/A | 2 | 2 | 1 | 0 | 2 | 2 | 2 | 0.82 |
| Eckhardt et al. (53) | 2015 | 2 | 2 | 2 | 2 | N/A | N/A | N/A | 2 | 2 | 2 | 2 | 2 | 2 | 2 | 1.00 |
| Engbers et al. (54) | 2007 | 2 | 2 | 2 | 2 | N/A | N/A | N/A | 2 | 2 | 2 | 2 | 2 | 2 | 2 | 1.00 |
| Engelen et al. (55) | 2017 | 2 | 2 | 2 | 0 | N/A | N/A | N/A | 2 | 2 | 2 | 2 | 2 | 2 | 2 | 0.91 |
| Eves & Masters (56) | 2006 | 0 | 2 | 2 | 2 | N/A | N/A | N/A | 2 | 2 | 2 | 2 | 2 | 2 | 2 | 0.91 |
| Eves et al. (58) | 2012 | 2 | 2 | 2 | 0 | 2 | N/A | N/A | 2 | 2 | 2 | 2 | 2 | 2 | 2 | 0.92 |
| Eves et al. (57) | 2012 | 2 | 2 | 2 | 0 | N/A | N/A | N/A | 2 | 2 | 2 | 2 | 2 | 2 | 2 | 0.91 |
| Ford & Torok (59) | 2010 | 0 | 2 | 2 | 1 | N/A | N/A | N/A | 2 | 2 | 1 | 0 | 1 | 1 | 2 | 0.64 |
| Garland et al. (60) | 2013 | 2 | 2 | 1 | 2 | N/A | N/A | N/A | 2 | 0 | 2 | 0 | 0 | 2 | 2 | 0.68 |
| Graham et al. (61) | 2013 | 2 | 2 | 2 | 2 | 1 | N/A | N/A | 2 | 2 | 2 | 2 | 2 | 2 | 2 | 0.96 |
| Grimstvedt et al. (62) | 2010 | 2 | 2 | 2 | 2 | N/A | N/A | N/A | 2 | 2 | 2 | 2 | 2 | 2 | 2 | 1.00 |
| Hodgin & Graham (63) | 2016 | 2 | 2 | 2 | 2 | 2 | 0 | N/A | 2 | 1 | 2 | 2 | 2 | 2 | 2 | 0.88 |
| Kerr et al. (64) | 2004 | 2 | 2 | 2 | 1 | N/A | N/A | N/A | 2 | 2 | 2 | 2 | 0 | 2 | 2 | 0.86 |
| Kerr et al. (65) | 2001 | 2 | 1 | 2 | 0 | N/A | N/A | N/A | 2 | 2 | 2 | 2 | 2 | 2 | 2 | 0.86 |
| Kerr et al. (66) | 2001 | 1 | 2 | 2 | 0 | N/A | N/A | N/A | 2 | 2 | 2 | 2 | 2 | 2 | 2 | 0.86 |
| Kerr et al. (67) | 2001 | 1 | 1 | 2 | 0 | N/A | N/A | N/A | 2 | 2 | 2 | 2 | 2 | 2 | 2 | 0.82 |
| Kerr et al. (68) | 2001 | 2 | 1 | 2 | 2 | N/A | N/A | N/A | 1 | 2 | 2 | 2 | 2 | 2 | 2 | 0.91 |
| Kwak et al. (69) | 2007 | 2 | 2 | 2 | 1 | N/A | N/A | N/A | 2 | 2 | 2 | 2 | 2 | 2 | 2 | 0.95 |
| Lewis & Eves (70) | 2012 | 2 | 2 | 2 | 1 | N/A | N/A | N/A | 2 | 2 | 2 | 2 | 2 | 2 | 2 | 0.95 |
| Lewis & Eves (71) | 2012 | 1 | 2 | 2 | 1 | N/A | N/A | N/A | 2 | 2 | 2 | 2 | 2 | 2 | 2 | 0.91 |
| Lewis & Eves (72) | 2011 | 2 | 2 | 2 | 1 | N/A | N/A | N/A | 2 | 2 | 2 | 2 | 2 | 2 | 2 | 0.95 |
| Marshall et al. (73) | 2002 | 2 | 2 | 2 | 0 | N/A | N/A | N/A | 2 | 2 | 2 | 2 | 2 | 2 | 2 | 0.91 |
| Moloughney et al. (74) | 2018 | 2 | 2 | 2 | 0 | N/A | N/A | N/A | 2 | 2 | 2 | 2 | 1 | 2 | 2 | 0.86 |
| Müller-Riemenschneider et al. (75) | 2010 | 2 | 2 | 2 | 1 | N/A | N/A | N/A | 2 | 2 | 2 | 2 | 2 | 2 | 2 | 0.95 |
| Olander & Eves (76) | 2011 | 2 | 2 | 2 | 1 | N/A | N/A | N/A | 2 | 2 | 2 | 2 | 2 | 2 | 2 | 0.95 |
| Olander et al. (77) | 2008 | 1 | 2 | 2 | 1 | N/A | N/A | N/A | 2 | 2 | 2 | 2 | 2 | 2 | 2 | 0.91 |
| Puig, Ribera & Eves (78) | 2009 | 2 | 2 | 2 | 2 | N/A | N/A | N/A | 2 | 2 | 2 | 2 | 2 | 2 | 2 | 1.00 |
| Slaunwhite et al. (79) | 2009 | 2 | 2 | 2 | 1 | N/A | N/A | N/A | 2 | 2 | 2 | 2 | 2 | 2 | 2 | 0.95 |
| Swenson & Siegel (80) | 2012 | 2 | 2 | 2 | 0 | N/A | N/A | N/A | 1 | 2 | 2 | 2 | 0 | 2 | 2 | 0.77 |
| Vanden Auweele et al. (81) | 2005 | 2 | 2 | 2 | 1 | N/A | N/A | N/A | 2 | 2 | 2 | 0 | 0 | 2 | 2 | 0.77 |
| Webb & Cheng (82) | 2010 | 2 | 1 | 2 | 2 | N/A | N/A | N/A | 2 | 2 | 2 | 2 | 2 | 2 | 2 | 0.95 |
| Webb & Eves (83) | 2007 | 2 | 2 | 2 | 2 | N/A | N/A | N/A | 2 | 2 | 2 | 2 | 2 | 2 | 2 | 1.00 |
| Webb & Eves (84) | 2007 | 2 | 2 | 2 | 2 | N/A | N/A | N/A | 2 | 2 | 2 | 2 | 2 | 2 | 2 | 1.00 |
| Webb & Eves (85) | 2005 | 2 | 1 | 2 | 2 | N/A | N/A | N/A | 1 | 2 | 2 | 2 | 2 | 2 | 2 | 0.91 |
| Arora et al. (86) | 2006 | 2 | 2 | 1 | 2 | 0 | 0 | 0 | 2 | 2 | 2 | 0 | 2 | 2 | 2 | 0.68 |
| Berenbaum & Cheung (87) | 2014 | 1 | 2 | 2 | 2 | 1 | 0 | 0 | 2 | 2 | 2 | 2 | 2 | 2 | 2 | 0.79 |
| Cho et al. (88) | 2018 | 2 | 2 | 2 | 2 | 0 | 0 | 3 | 2 | 2 | 2 | 0 | 2 | 2 | 1 | 0.73 |
| Cohen et al. (89) | 2017 | 2 | 2 | 2 | 2 | 1 | 0 | 0 | 2 | 1 | 2 | 0 | 2 | 2 | 2 | 0.71 |
| Daffu-O'Reilly et al. (90) | 2017 | 2 | 2 | 2 | 2 | 2 | N/A | 2 | 2 | 2 | 2 | 0 | 2 | 2 | 2 | 0.92 |
| De Bruijn et al. (91) | 2014 | 2 | 2 | 1 | 2 | 1 | 0 | 0 | 2 | 2 | 2 | 0 | 2 | 2 | 2 | 0.71 |
| Gray et al. (92) | 2011 | 2 | 2 | 2 | 2 | 1 | 0 | 0 | 2 | 2 | 2 | 2 | 2 | 2 | 2 | 0.82 |
| Jones et al. (93) | 2004 | 2 | 2 | 2 | 2 | 1 | 0 | 0 | 2 | 2 | 2 | 0 | 0 | 2 | 2 | 0.68 |
| Jones et al. (94) | 2003 | 1 | 2 | 2 | 2 | 1 | 0 | 0 | 2 | 2 | 2 | 0 | 2 | 2 | 2 | 0.71 |
| Kozak et al. (95) | 2013 | 2 | 2 | 2 | 2 | 2 | 0 | 0 | 2 | 0 | 2 | 0 | 2 | 2 | 2 | 0.71 |
| Latimer et al. (96) | 2008 | 2 | 2 | 2 | 2 | 2 | 0 | 0 | 2 | 2 | 2 | 2 | 2 | 2 | 2 | 0.86 |
| Li et al. (97) | 2017 | 2 | 1 | 2 | 2 | 1 | 0 | 0 | 2 | 2 | 2 | 0 | 2 | 2 | 2 | 0.71 |
| Li et al. (98) | 2013 | 2 | 2 | 2 | 2 | 1 | 0 | 0 | 2 | 2 | 2 | 1 | 2 | 2 | 2 | 0.79 |
| Lithopoulos & Young (99) | 2016 | 2 | 1 | 2 | 2 | 1 | 0 | 0 | 2 | 2 | 2 | 2 | 2 | 2 | 2 | 0.79 |
| McCall et al. (100) | 2004 | 2 | 2 | 2 | 2 | 1 | 0 | 0 | 2 | 1 | 2 | 0 | 2 | 2 | 2 | 0.71 |
| Morris et al. (101) | 2016 | 2 | 1 | 2 | 2 | 1 | 0 | 0 | 2 | 2 | 2 | 0 | 1 | 2 | 2 | 0.68 |
| Notthoff et al. (102) | 2016 | 2 | 1 | 2 | 2 | N/A | N/A | N/A | 2 | 2 | 2 | 2 | 2 | 2 | 2 | 0.95 |
| Notthoff & Carstensen (103) | 2014 | 2 | 2 | 2 | 2 | 1 | 0 | 0 | 2 | 1 | 2 | 2 | 2 | 2 | 2 | 0.79 |
| Ratcliff et al. (104) | 2019 | 2 | 2 | 2 | 2 | 1 | 0 | 0 | 2 | 2 | 2 | 1 | 2 | 2 | 2 | 0.79 |
| Vanroy et al. (105) | 2019 | 2 | 2 | 2 | 2 | 2 | 3 | 3 | 2 | 1 | 2 | 2 | 2 | 2 | 2 | 0.96 |
| Van 't Riet et al. (106) | 2010 | 2 | 2 | 1 | 2 | 2 | 0 | 0 | 2 | 2 | 2 | 0 | 2 | 2 | 2 | 0.75 |
| Wirtz & Kulpavaropas (107) | 2014 | 2 | 2 | 2 | 2 | 1 | 0 | 0 | 2 | 1 | 2 | 0 | 0 | 2 | 2 | 0.64 |
| Zenko et al. (108) | 2016 | 2 | 1 | 2 | 2 | 1 | 0 | 0 | 2 | 2 | 2 | 0 | 2 | 2 | 2 | 0.71 |
| Cooley et al. (109) | 2008 | 2 | 2 | 2 | 0 | N/A | N/A | N/A | 2 | 2 | 2 | 2 | 1 | 2 | 2 | 0.86 |
| King et al. (38) | 2016 | 2 | 2 | 2 | 2 | 2 | 2 | N/A | 2 | 2 | 2 | 2 | 2 | 2 | 2 | 1.00 |
| Van Hoecke et al. (110) | 2017 | 1 | 2 | 2 | 0 | N/A | N/A | N/A | 2 | 2 | 2 | 0 | 1 | 2 | 2 | 0.73 |
| Gorin et al. (111) | 2013 | 2 | 2 | 2 | 2 | 1 | 0 | N/A | 2 | 2 | 2 | 2 | 2 | 2 | 2 | 0.88 |
| Van Calster et al. (112) | 2017 | 2 | 2 | 2 | 2 | N/A | N/A | N/A | 2 | 2 | 2 | 0 | 1 | 2 | 2 | 0.86 |
| Zhang et al. (113) | 2015 | 2 | 2 | 2 | 2 | 2 | 2 | N/A | 2 | 2 | 2 | 2 | 2 | 2 | 2 | 1.00 |
| Howie et al. (114) | 2011 | 2 | 2 | 2 | 0 | N/A | N/A | N/A | 2 | 2 | 2 | 0 | 2 | 2 | 2 | 0.82 |
| Patel et al. (115) | 2019 | 2 | 2 | 2 | 2 | 2 | 2 | 3 | 2 | 2 | 2 | 2 | 2 | 2 | 2 | 1.00 |
| Tullar et al. (116) | 2019 | 2 | 2 | 2 | 2 | 3 | 3 | 3 | 1 | 2 | 2 | 2 | 2 | 2 | 2 | 0.95 |
| Zhang et al. (117) | 2016 | 2 | 2 | 2 | 2 | 2 | 2 | 2 | 2 | 2 | 2 | 2 | 2 | 2 | 2 | 1.00 |
| Patel et al. (118) | 2017 | 2 | 2 | 2 | 2 | 2 | 2 | N/A | 2 | 2 | 2 | 2 | 2 | 2 | 2 | 1.00 |
| Strath et al. (119) | 2011 | 2 | 2 | 2 | 2 | 2 | 0 | 0 | 2 | 0 | 2 | 2 | 1 | 2 | 2 | 0.75 |
| Anson et al. (120) | 2016 | 2 | 2 | 2 | 2 | 2 | 2 | 1 | 2 | 1 | 2 | 0 | 2 | 2 | 2 | 0.86 |
| Venema et al. (121) | 2017 | 2 | 2 | 1 | 2 | N/A | N/A | N/A | 2 | 2 | 2 | 2 | 2 | 2 | 2 | 0.95 |

Note. Items correspond to the QualSyst tool for quantitative studies [1]. Rating: 2 = Yes; 1 = Partial; 0 = No; N/A = Not applicable.

**References**

Kmet, L.M., L.S. Cook, and R.C. Lee, *Standard quality assessment criteria for evaluating primary research papers from a variety of fields*. 2004.
